# Genome-wide haplotype association analysis of primary biliary cholangitis risk in Japanese

**DOI:** 10.1038/s41598-018-26112-1

**Published:** 2018-05-17

**Authors:** Cindy Im, Yadav Sapkota, Wonjong Moon, Minae Kawashima, Minoru Nakamura, Katsushi Tokunaga, Yutaka Yasui

**Affiliations:** 1 School of Public Health, University of Alberta, Edmonton, Alberta T6G 1C9 Canada; 2 Department of Epidemiology and Cancer Control, St. Jude Children’s Research Hospital, Memphis, TN 38105 USA; 3 Department of Human Genetics, Graduate School of Medicine, The University of Tokyo, Tokyo, 113-0033 Japan; 4 Department of Hepatology, Nagasaki University Graduate School of Biomedical Sciences and Clinical Research Center, National Hospital Organization Nagasaki Medical Center, Omura, Nagasaki, 856-8562 Japan

## Abstract

Primary biliary cholangitis (PBC) susceptibility loci have largely been discovered through single SNP association testing. In this study, we report genic haplotype patterns associated with PBC risk genome-wide in two Japanese cohorts. Among the 74 genic PBC risk haplotype candidates we detected with a novel methodological approach in a discovery cohort of 1,937 Japanese, nearly two-thirds were replicated (49 haplotypes, Bonferroni-corrected P < 6.8 × 10^−4^) in an independent Japanese cohort (N = 949). Along with corroborating known PBC-associated loci (*TNFSF15*, *HLA-DRA*), risk haplotypes may potentially model *cis*-interactions that regulate gene expression. For example, one replicated haplotype association (9q32–9q33.1, OR = 1.7, P = 3.0 × 10^−21^) consists of intergenic SNPs outside of the human leukocyte antigen (HLA) region that overlap regulatory histone mark peaks in liver and blood cells, and are significantly associated with *TNFSF8* expression in whole blood. We also replicated a novel haplotype association involving non-HLA SNPs mapped to *UMAD1* (7p21.3; OR = 15.2, P = 3.9 × 10^−9^) that overlap enhancer peaks in liver and memory T_h_ cells. Our analysis demonstrates the utility of haplotype association analyses in discovering and characterizing PBC susceptibility loci.

## Introduction

Primary biliary cholangitis (PBC; also known as primary biliary cirrhosis, MIM 109720) is a progressive autoimmune disease of the liver, leading to the destruction of the bile ducts, and in end-stage cases, liver failure. While the factors that underlie PBC susceptibility and increase risk for disease progression remain enigmatic^1,2^, a concordance rate of 63% for PBC has been observed in monozygotic twins, suggesting that PBC has a strong hereditary component^3^.

Recent genome-wide association studies (GWAS) in European cohorts have confirmed associations between the human leukocyte antigen (HLA) locus and identified over two dozen non-HLA PBC susceptibility loci^4–7^. PBC GWAS in Japanese cohorts have only replicated a minor number of risk loci identified in European populations (e.g., *IL7R*, *IKZF3*, *CD80*), and revealed features of the PBC genetic architecture that may be dissimilar between populations, with *TNFSF15*, *POU2AF1*, and *PRKCB* emerging as major susceptibility loci among Japanese^8,9^. While these results suggest that additional single-SNP GWAS with larger sample sizes are warranted, it is also worthwhile to consider complementary analytic approaches to gain further insights. One such approach involves the exploration of the effects of haplotypes, or the arrangement of multiple SNP alleles on the same chromosome. Haplotype patterns may not only be more powerful for mapping disease genes, but may also be uniquely informative about known single SNP associations, since haplotypes are also known to vary considerably between populations, bear signatures of selection, and may contextualize the role genetic variants play, singly or in tandem, in disease pathogenesis^10–12^.

Among existing methods to conduct genome-wide haplotype association studies with unphased genotype data, the most popular strategy is to split the genome indiscriminately into overlapping “sliding” windows, and simultaneously infer frequencies for all possible haplotypes among a small, fixed number of SNPs within each window and test global associations under a regression-based framework^11,13,14^. Due to the computational burden of simultaneously inferring and testing haplotypes, the selected windows for haplotype formation are small, and constructed haplotypes consider a limited number of contiguous SNPs. An important consequence of these analytic restrictions is that a comprehensive exploration of haplotypes as models for the *cis*-regulation of gene expression is typically not feasible, given that gene expression is modulated by haplotypes comprised of both proximal and distal SNPs with individual or interacting effects^15,16^ and may be influenced by larger networks of SNPs (i.e., three SNPs or more)^17^.

In the current study, we conducted a genome-wide gene-based haplotype association analysis to uncover relationships between SNPs that are potentially transcriptionally-relevant and are also associated with PBC risk. We applied a regression-based methodology to find haplotypes consisting of three SNPs associated with PBC risk among sets of SNPs mapped to extended gene-centered windows in a discovery cohort of 1,937 Japanese, without restricting formed 3-SNP haplotypes to contiguous SNPs. Phased haplotypes inferred with whole chromosome SNP data were treated as observed in our downstream association analysis. We used a permutation-based approach to select top haplotypes and replicated selected findings in a second independent Japanese cohort (N = 949). Ancillary bioinformatics analyses were conducted to assess the biological plausibility of associations between PBC risk and detected 3-SNP haplotypes.

## Results

### Discovery and replication of genic haplotypes associated with PBC risk

We developed a novel approach to detect haplotypes associated with PBC risk by leveraging the logic regression^18^ methodology, a statistical learning method that employs a stochastic search algorithm to detect combinations of binary predictors (i.e., haplotypes) associated with an outcome of interest (i.e., PBC risk) within a generalized linear model (GLM) framework. After computationally resolving haplotype phase for the 2,886 participants in the combined study cohort, a total of 272,131 SNPs were mapped to at least one of 15,137 gene analytic windows, or broadly-defined gene regions that include RefSeq genes and the flanking 500-kb regions before and after transcription start and stop sites (Supplementary Fig. S1). Using the proposed methodology, we identified the most strongly associated haplotype consisting of three SNPs for every gene analytic window in our discovery cohort (N = 1,937). A total of 7,317 unique candidate 3-SNP haplotype associations were detected: 7,272 genic haplotypes included no SNPs in the HLA region (chr6:29645000–33365000, hg19 build), while 45 genic haplotypes included at least one HLA region SNP.

**Figure 1:**
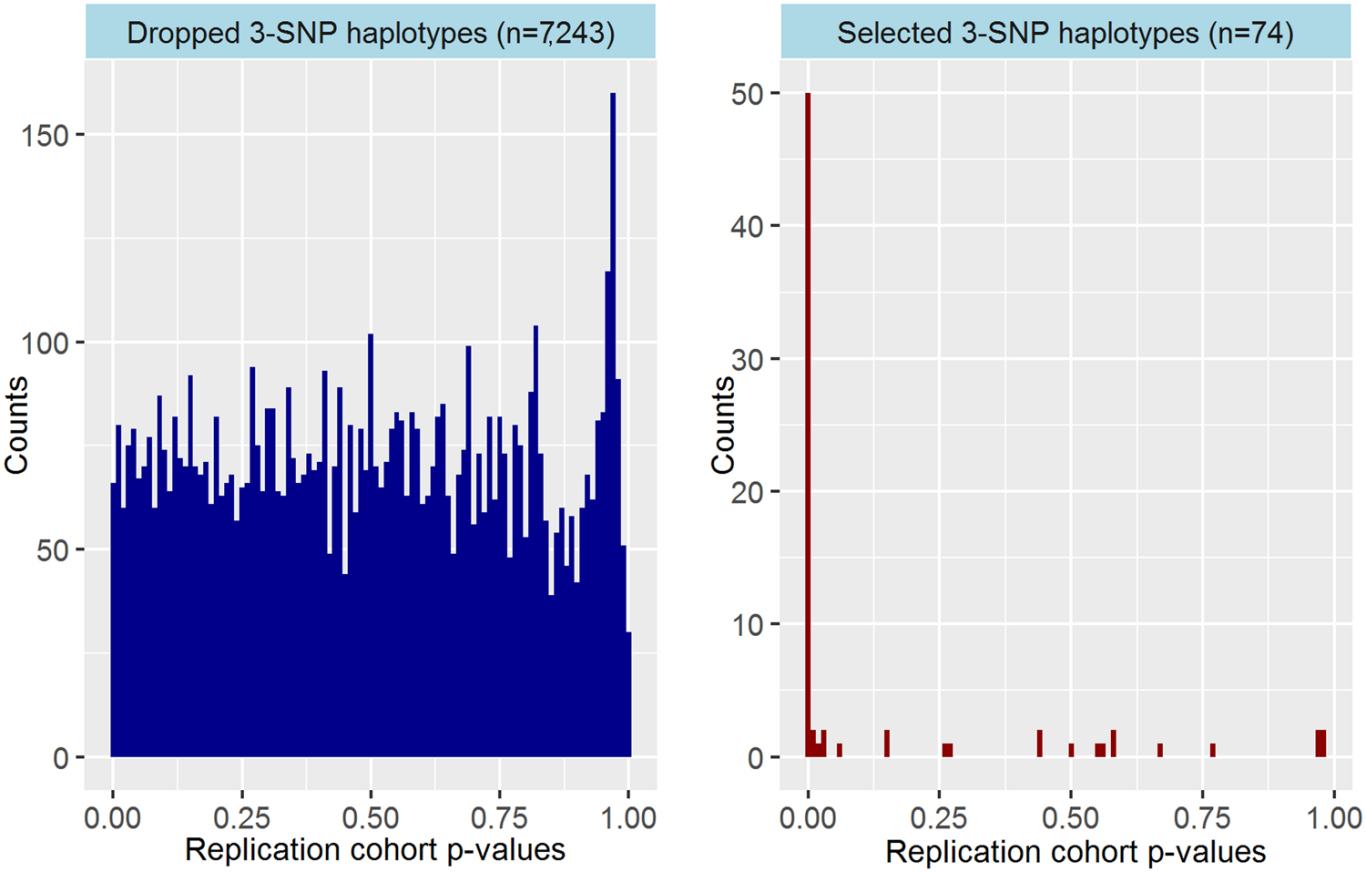
Distributions of haplotype association test p-values for dropped versus selected 3-SNP haplotypes in the replication cohort. Side-by-side histograms of haplotype association p-values for dropped haplotypes and selected haplotypes in our replication cohort (N = 949) are provided for comparison. Selected 3-SNP haplotypes have the top percentile of permutation-based evaluation statistics (values less than −11.4).

We used a permutation-based evaluation statistic^19^ to assess whether the candidate genic 3-SNP haplotypes had stronger associations with PBC risk than expected. Applying a pre-defined cut-off (top one percentile of permutation-based evaluation statistic values), 74 candidate 3-SNP haplotype associations were selected for replication follow-up. We employed a Bonferroni-corrected per-test significance threshold (P < 0.05/74 = 6.8 × 10^−4^) in our replication analysis with our independent cohort (N = 949). Under this p-value threshold, nearly two-thirds of selected 3-SNP haplotypes were replicated (49/74 haplotypes). All genic haplotypes with HLA region SNPs were replicated (37/37 trees), while 32.4% of genic haplotypes without HLA region SNPs (12/37 trees) were replicated. To contextualize the efficacy of our permutation-based evaluation statistic cut-off in capturing the strongest haplotype signals detected with logic regression, Fig. 1 contrasts the distribution of p-values for association tests in the replication cohort (N = 949) for the 74 selected 3-SNP haplotypes to the relatively uniform replication p-value distribution for the 7,243 dropped 3-SNP haplotypes.

Discovery and replication analysis results for the 49 replicated 3-SNP genic haplotypes are provided in Supplementary Table S1. Among replicated haplotypes, 69.4% (34) included at least one SNP that was not nominally significant, while 24.5% (12) contained no SNPs that individually achieved genome-wide significance (P < 5 × 10^−8^) (Supplementary Table S2). The magnitude of estimated ORs for replicated 3-SNP haplotypes in the combined cohort (N = 2,886) assuming additive haplotype effects ranged from 1.672 to 15.246 (inverting protective associations), with p-values ranging from 1.3 × 10^−35^ to 3.9 × 10^−9^. Tables 1 and 2 highlight selected example results for five replicated 3-SNP haplotypes. Among these examples, (rs9295704 = C) and ((rs2451752 = A) and (rs2575174 = C)) on chromosome 6 (OR = 0.365, P = 8.9 × 10^−15^), and ((rs12671658 = T) or (rs12702656 = A)) and (rs11768586 = G) on chromosome 7 (OR = 0.066, P = 3.9 × 10^−9^) represent novel non-HLA loci associated with PBC risk (Table 2). Other examples provided correspond to genomic regions carrying single variants with the strongest associations with PBC risk in previous PBC GWAS in Japanese (near *HLA-DRA* and *TNFSF15*)^8,9^.

**Table 1:** Selected examples of replicated 3-SNP haplotypes.

Chr	3-SNP haplotype or logic tree	Gene analytic windows with 3-SNP haplotype	# SNPs in gene windows	Discovery (N = 1,937)			Replication (N = 949)		Combined (N = 2,886)				
				Permutation-based selection statistic	OR	P	OR	P	OR	P	# with 0 haplotype copies (% cases)	# with 1 haplotype copy (% cases)	# with 2 haplotype copies (% cases)
6	(rs3129881 = C) or ((rs375244 = A) and (rs3132947 = G))	*CFB; NELFE; C2; C2-AS1*	153–163	−69.730	4.937	1.8 × 10^−20^	2.324	1.6 × 10^−5^	3.665	2.3 × 10^−24^	17 (17.6%)	360 (21.7%)	2,509 (51.8%)
6	((rs9268831 = T) or (rs9269190 = T)) or (rs9270652 = C)	*ATF6B; FKBPL; PPT2; PPT2-EGFL8; AGPAT1*	158–169	−43.565	3.640	3.2 × 10^−23^	2.315	1.9 × 10^−7^	3.075	7.3 × 10^−29^	46 (15.2%)	496 (26.0%)	2,344 (53.1%)
6	(rs9295704 = C) and ((rs2451752 = A) and (rs2575174 = C))^a^	*BTN3A2; BTN2A2*	46–52	−12.263	0.362	1.7 × 10^−10^	0.374	1.3 × 10^−5^	0.365	8.9 × 10^−15^	2,571 (50.4%)	304 (27.3%)	11 (9.1%)
7	((rs12671658 = T) or (rs12702656 = A)) and (rs11768586 = G)^a^	*GLCCI1; LOC100505921; ICA1; COL28A1; MIOS; RPA3; LOC100505938; UMAD1; LOC101927391*	204–292	−16.424	0.040	8.7 × 10^−6^	0.112	3.6 × 10^−4^	0.066	3.9 × 10^–9^	2,802 (49.1%)	84 (6.0%)	0 (NA)
9	(rs4979484 = C) or ((rs13300483 = T) and (rs7028891 = G))^a^	*LOC100505478; TNFSF15; C9orf91; TNFSF8*	121–156	−28.431	1.746	4.2 × 10^−17^	1.528	8.6 × 10^−6^	1.672	3.0 × 10^−21^	775 (35.4%)	1,434 (48.7%)	677 (60.4%)

Abbreviation: #, number.

^a^Contains no HLA region SNPs.

**Table 2:** Single SNP and component 2-SNP haplotype effects for example replicated 3-SNP haplotypes.

Chr	3-SNP haplotype or logic tree	Tree OR^b^	Tree P^b^	Single SNP	Alternative allele	SNP OR^c^	SNP P^c^	Component 2-SNP haplotype	Pair OR^d^	Pair P^d^
6	(rs3129881 = C) or ((rs375244 = A) and (rs3132947 = G))	3.665	2.3 × 10^−24^	rs3129881	C	2.227	1.8 × 10^−23^	rs375244 = A and rs3132947 = G	1.238	7.9 × 10^−5^
				rs375244	A	0.980	0.715			
				rs3132947	G	2.117	1.4 × 10^−16^			
6	((rs9268831 = T) or (rs9269190 = T)) or (rs9270652 = C)	3.075	7.3 × 10^−29^	rs9268831	T	1.324	1.6 × 10^−7^	rs9268831 = T or rs9269190 = T	1.633	2.8 × 10^−16^
				rs9269190	T	1.252	1.3 × 10^−4^	rs9268831 = T or rs9270652 = C	1.321	2.0 × 10^−5^
				rs9270652	C	1.109	0.057	rs9269190 = T or rs9270652 = C	1.925	1.2 × 10^−18^
6	(rs9295704 = C) and ((rs2451752 = A) and (rs2575174 = C))^a^	0.365	8.9 × 10^−15^	rs9295704	C	0.669	1.5 × 10^−7^	rs9295704 = C and rs2451752 = A	0.399	3.2 × 10^−14^
				rs2451752	A	0.951	0.457	rs9295704 = C and rs2575174 = C	0.638	1.2 × 10^−7^
				rs2575174	C	0.940	0.393	rs2451752 = A and rs2575174 = C	0.933	0.228
7	((rs12671658 = T) or (rs12702656 = A)) and (rs11768586 = G)^a^	0.066	3.9 × 10^−9^	rs12671658	T	1.023	0.682	rs12671658 = T and rs11768586 = G	0.104	1.4 × 10^−6^
				rs12702656	A	1.039	0.585	rs12702656 = A and rs11768586 = G	0.000	0.953
				rs11768586	G	0.865	0.010			
9	(rs4979484 = C) or ((rs13300483 = T) and (rs7028891 = G))^a^	1.672	3.0 × 10^−21^	rs4979484	C	1.365	0.005	rs13300483 = T and rs7028891 = G	1.637	1.2 × 10^−19^
				rs13300483	T	1.584	1.4 × 10^−17^			
				rs7028891	G	1.574	2.8 × 10^−17^			

^a^Contains no HLA region SNPs.

^b^3-SNP haplotype OR and p-value in the combined sample (N = 2,886).

^c^Single SNP ORs and p-values, assuming an additive genetic effect model for the specified alternative allele.

^d^2-SNP haplotype ORs and p-values, assuming an additive genetic effect model for the specified haplotype pattern.

Missingness rates for replicated 3-SNP haplotypes in the unphased data among all available controls (median: 1.13%, IQR: 0.86%; N = 1,505) and cases (median: 1.09%, IQR: 0.80%; N = 1,381) were low and comparable between groups (Supplementary Table S3). Pre-phasing counts of unambiguous homozygous carriers and potential carriers of at least one haplotype copy were consistent with the estimated corresponding replicated haplotype counts in the phased data for all available controls (N = 1,505, Supplementary Table S4). Lastly, frequency distributions for each of the replicated 3-SNP haplotypes among all phased study controls (N = 1,505) and the phased 1000G JPT reference panel (N = 104) demonstrated that these distributions were comparable for each haplotype (Supplementary Table S4).

### Comparing proposed and benchmark methodologies for haplotype detection

We applied a benchmark haplotype association methodology to conduct global tests of association^14^ for estimated haplotypes formed within all available sliding window sets comprised of three contiguous SNPs in each of the 15,137 gene analytic windows in our discovery cohort (N = 1,937). Using this benchmark method, we identified 1,425 haplotypes with p-values meeting a Bonferroni-corrected p-value threshold (P < 0.05/1,567,361 tests = 3.2 × 10^−8^) across 205 gene analytic windows (Table 3). Nearly two-thirds of the gene windows (135/205) with a top haplotype association detected by the benchmark method was a window that also contained a replicated 3-SNP haplotype detected with our proposed method, suggesting the proposed and benchmark methods found many of the same gene analytic windows to be important for haplotype exploration. No exact matches for 3-SNP haplotypes detected by logic regression were observed among top haplotypes identified by the benchmark method.

**Table 3:** Comparison of logic regression and benchmark methods to detect 3-SNP haplotypes in the discovery cohort, N = 1,937.

Chr	Method A (proposed): Logic regression			Method B (benchmark): 3-SNP sliding windows				Comparison
	# Gene windows with replicated 3-SNP haplotype (Method A)	Gene window with best p-value	Best p-value	# Tests with P < 3.2 × 10^−8^ (Bonferroni)	# Gene windows with at least one haplotype with P < 3.2 × 10^−8^	Gene window with best p-value	Best p-value	# Gene window matches between methods A and B
2	0	NA	NA	1	1	*LRP1B*	1.1 × 10^−9^	0
3	0	NA	NA	5	3	*NEK10*	3.6 × 10^−18^	0
6	143	*NOTCH4*	6.1 × 10^−27^	1352	173	*TAAR2*	2.2 × 10^−30^	123
7	9	*GLCCI1*	8.7 × 10^−6^	6	3	*HGF*	2.1 × 10^−15^	0
8	0	NA	NA	4	4	*CYP11B1*	1.1 × 10^−8^	0
9	12	*DEC1*	2.5 × 10^−17^	74	16	*DEC1*	3.2 × 10^−13^	12
18	0	NA	NA	10	5	*MTCL1*	1.6 × 10^−10^	0

Abbreviation: #, number.

Given that SNPs contributing to 3-SNP haplotypes detected by logic regression are not necessarily contiguous and estimated haplotypes were treated as observed in this analysis, we computed global test score statistics with variance estimates that account for the uncertainty in the haplotype estimation^14^ for estimated haplotypes consisting of the three SNPs in each of the replicated 3-SNP haplotype trees in the combined cohort (N = 2,886). The scale of each haplotype tree’s global test p-value appeared to be consistent with the haplotype p-value obtained with the proposed method (Supplementary Table S5).

### Functional annotation of replicated haplotype SNPs

**Figure 2:**
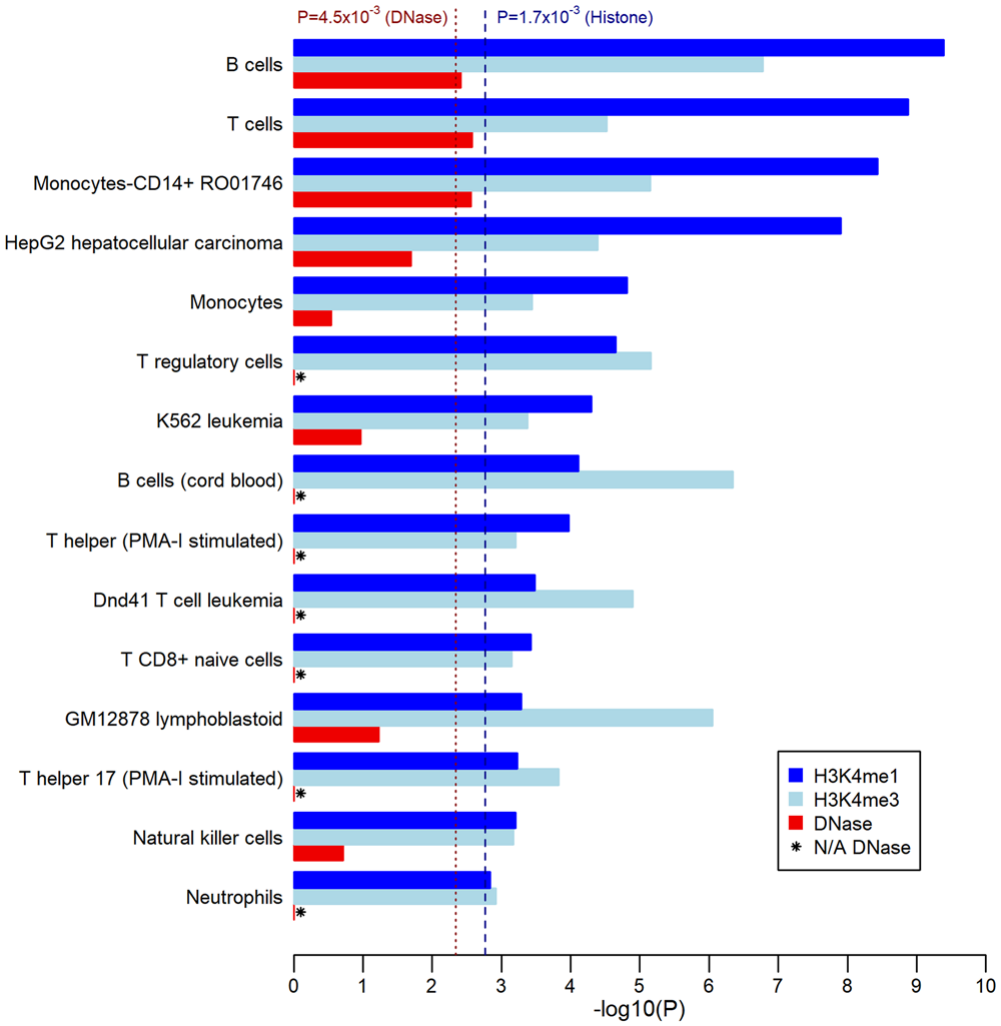
Selection of histone modification and DNase peak enrichment analysis results. The enrichment analyses compared 106 SNPs in replicated 3-SNP haplotypes to nominally associated single SNPs in gene regions. Figure shows enrichment test p-values that are log-transformed (-log_10_(P)) for the 15 blood/liver cell types for which haplotype SNPs are significantly enriched for both H3K4me1 and H3K4me3 peaks. Dashed and dotted vertical lines show Bonferroni-corrected p-value thresholds based on the number of blood/liver cell types for which Roadpmap Epigenomics assay data was available (29 and 11 types for histone mark and DNase peaks, respectively).

We conducted enrichment analyses to broadly investigate the biological plausibility of replicated 3-SNP haplotypes’ associations with PBC risk by comparing the set of 106 unique SNPs contributing to replicated 3-SNP haplotypes (“haplotype SNPs”) against an unpruned comparison set of 16,036 SNPs mapped to gene analytic windows with nominal univariate associations with PBC risk (P < 0.05). An examination of the number of gene expressions (eQTLs) significantly associated with haplotype SNPs in three blood and liver cell/tissue types (Genotype-Tissue Expression, GTEx v7)^20^ demonstrated that haplotype SNPs were significantly enriched for eQTLs in lymphoblastoid cells relative to the comparison SNP set (P = 2.7 × 10^−3^) (Supplementary Table S6). The set of haplotype SNPs was also significantly enriched for overlaps with ChIP-seq histone modification peaks linked with enhancer (H3K4me1) or promoter (H3K4me3) activity in at least 20 of the 29 consolidated blood and liver cell types available in Roadmap Epigenomics Mapping Consortium^21^ (REMC) data (Bonferroni-corrected P < 0.05/29 = 1.7 × 10^−3^; Supplementary Tables S7, S8), respectively. Haplotype SNPs were also enriched for an indicator of open chromatin (DNase I peaks) in four blood cell types among 11 blood/liver cell types with REMC assay data (Bonferroni-corrected P < 0.05/11 = 4.5 × 10^−3^; Supplementary Table S9). Haplotype SNPs were jointly enriched for enhancer and promoter peaks in 15 blood/liver cell types, and simultaneously enriched for all three chromatin state indicators in three cell types in peripheral blood: primary B cells, primary T cells, and monocytes (Fig. 2). These results are consistent with reported immune-related PBC disease mechanisms that implicate B cell and T cell differentiation pathways^22^, and observations of significant inflammatory cell infiltration (including B cells, T cells, and macrophages) associated with the loss of biliary epithelial cells in the portal tract^23^.

**Figure 3:**
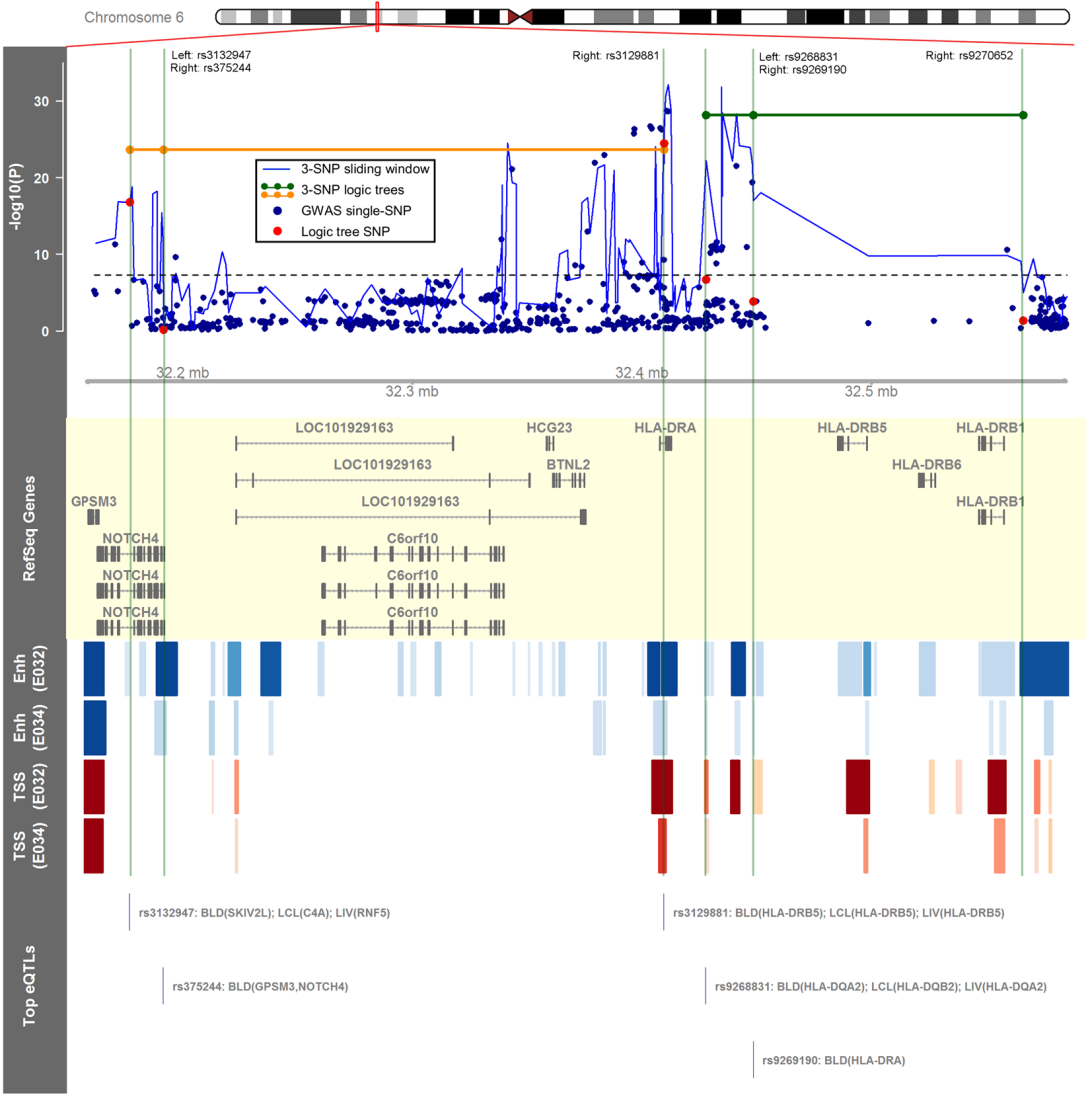
Visualization of two replicated 3-SNP haplotype logic trees containing SNPs in the HLA region. The plotted region spans chr6:32156782–32585905 (hg19), which corresponds with the red rectangle in the chromosomal ideogram. The top data track shows single-SNP association results and haplotype association results using benchmark (“3-SNP sliding window”) and proposed (“3-SNP logic tree”) methods for the selected genomic region. The subsequent annotation tracks show RefSeq genes, a heatmap corresponding to Roadmap Epigenomics^21^ H3K4me1 (Enh) and H3K4me3 (TSS) ChIP-seq peaks in primary B cells (E032) and primary T cells (E034), and the top significant blood (BLD), lymphoblastoid (LCL), and liver (LIV) eQTLs (expression quantitative trait loci) associated with SNPs in replicated haplotypes (GTEx Consortium)^20^.

Figure 3 highlights two replicated 3-SNP haplotypes in the HLA region near rs3129887, the variant with the strongest single-SNP association with PBC risk in Japanese^8,9^. Association testing results for haplotypes detected with logic regression and the benchmark method are shown in the top data track in Fig. 3 across chr6:32156782–32585905, followed by a visual summary of relevant functional annotations for SNPs in the replicated 3-SNP haplotypes. One of the replicated 3-SNP haplotypes shown is (rs3129881 = C) or ((rs375244 = A) and (rs3132947 = G)) (OR = 3.665, P = 2.3 × 10^−24^), with SNPs in *NOTCH4* and *HLA-DRA* introns; the other is ((rs9268831 = T) or (rs9269190 = T)) or (rs9270652 = C) (OR = 3.075, P = 7.3 × 10^−29^), comprised of intergenic SNPs near *HLA-DRA* and *HLA-DRB1*. These haplotypes connect SNPs that may be linearly far apart, can consist of SNPs that do not achieve genome-wide significance (P < 5 × 10^−8^), and combine SNPs across multiple top 3-SNP haplotype windows tagged by the benchmark method. Additionally, all six haplotype SNPs overlap H3K4me1 and/or H3K4me3 peaks in primary B or T cells, while five SNPs have at least one significant eQTL in whole blood, lymphoblastoid, or liver cells.

Replicated 3-SNP haplotypes in chromosomes 7 and 9 include SNPs that overlap genomic regions tagged with the smallest p-values identified by the benchmark method across chr7:7667281–8026742 and chr9:116727079–118438852, respectively, while corresponding functional annotations contextualize the relative contributions of each SNP (Supplementary Figs S2 and S3). To clarify, two intergenic SNPs in the chromosome 9 (rs4979484 = C) or ((rs13300483 = T) and (rs7028891 = G)) haplotype with strong univariate associations with PBC risk (P < 5 × 10^−8^) also have significant associations with *TNFSF8* expression in whole blood. However, this haplotype also includes rs4979484, an intergenic SNP with a relatively weak marginal association with PBC risk (P = 0.005) (Table 2). Interestingly, rs4979484 not only overlaps H3K4me1 and H3K4me3 peaks in multiple blood/liver cell types, but is also in a region with evidence of binding to nuclear factor kappa B (NF-kB), a transcription factor reported to play a critical role in inflammation and immunity processes^24^, in a lymphoblastoid cell line of Japanese origin (GM18951; Table 4, Supplementary Table S10).

**Table 4:** Functional annotations of SNPs in selected replicated 3-SNP haplotypes.

Chr	3-SNP haplotype	SNP	Chr position (hg19)	Ontology	Mapped gene	DHS overlap^a^, # EIDs (# PBC EIDs)	H3K4me1 overlap^a^, # EIDs (# PBC EIDs)	H3K4me3 overlap^a^, # EIDs (# PBC EIDs)	Bound protein^b^: Cell line (protein)	# Altered motifs^c^	Significant eQTLs^d^: Tissue (gene)
6	(rs3129881 = C) or ((rs375244 = A) and (rs3132947 = G))	rs3129881	32409484	intronic	*HLA-DRA*	0 (0)	42 (20)	50 (17)	GM12878 (OCT2, POL2, POL24H8, POU2F2); GM12891 (OCT2, POL2, POL24H8, POU2F2); GM12892 (POL2, POL24H8)	3	Whole Blood (*C4A, C4B, HLA-DQA1, HLA-DQA2, HLA-DQB2, HLA-DRB5*); Lymphoblastoid (*HLA-DQA2, HLA-DRB5*); Liver (*HLA-DRB5*)
		rs375244	32191457	intronic	*NOTCH4*	0 (0)	69 (15)	38 (3)	NA	3	Whole Blood (*GPSM3, NOTCH4*)
		rs3132947	32176782	intronic	*NOTCH4*	0 (0)	2 (1)	4 (0)	NA	3	Whole Blood (*AGPAT1, C4A, C4B, CYP21A1P, HLA-DRA, PBX2, SKIV2L*); Lymphoblastoid (*C4A, HLA-DQA1, RNF5*); Liver (*AGPAT1, RNF5*)
6	((rs9268831 = T) or (rs9269190 = T)) or (rs9270652 = C)	rs9268831	32427748	intergenic	*HLA-DRA* (dist = 14922), *HLA-DRB5* (dist = 57406)	5 (1)	31 (19)	71 (16)	GM18951 (POL2)	1	Whole Blood (*HLA-DQA1, HLA-DQA2, HLA-DQB1, HLA-DQB1-AS1, HLA-DQB2, HLA-DRB1, HLA-DRB6, HLA-DRB9*); Lymphoblastoid (*HLA-DQA2, HLA-DQB2, HLA-DRB6, HLA-DRB9, NOTCH4*); Liver (*HLA-DQA2, HLA-DQB2*)
		rs9269190	32448500	intergenic	*HLA-DRA* (dist = 35674), *HLA-DRB5* (dist = 36654)	2 (2)	4 (4)	2 (2)	NA	2	Whole Blood *(HLA-DRA*)
		rs9270652	32565905	intergenic	*HLA-DRB1* (dist = 8292), *HLA-DQA1* (dist = 39278)	1 (1)	1 (1)	2 (0)	NA	0	NA
6	(rs9295704 = C) and ((rs2451752 = A) and (rs2575174 = C))	rs9295704^e^	26704816	intergenic	*ZNF322* (dist = 44836), *GUSBP2* (dist = 134450)	0 (0)	13 (1)	8 (1)	NA	4	Whole Blood (*ABT1*)
		rs2451752^e^	26648013	intronic	ZNF322	0 (0)	1 (1)	3 (1)	NA	0	Whole Blood (*BTN3A1, BTN3A2, HMGN4, ZNF322*)
		rs2575174^e^	25885552	intergenic	*SLC17A3* (dist = 11081), *SLC17A2* (dist = 27432)	0 (0)	5 (0)	0 (0)	NA	2	Whole Blood (*HIST1H1T, HIST1H4A*)
7	((rs12671658 = T) or (rs12702656 = A)) and (rs11768586 = G)	rs12671658^e^	7842281	intronic	*UMAD1*	1 (0)	6 (0)	2 (0)	NA	7	NA
		rs12702656^e^	7851742	intronic	*UMAD1*	0 (0)	9 (3)	0 (0)	NA	4	NA
		rs11768586^e^	7849806	intronic	*UMAD1*	1 (0)	6 (2)	1 (1)	NA	0	NA
9	(rs4979484 = C) or ((rs13300483 = T) and (rs7028891 = G))	rs4979484^e^	117751450	intergenic	*TNFSF8* (dist = 58575), *TNC* (dist = 30404)	22 (6)	33 (20)	11 (7)	GM12878 (BATF, NFKB); GM12891 (NFKB); GM15510 (NFKB); GM18951 (NFKB)	3	NA
		rs13300483^e^	117643362	intergenic	*TNFSF15* (dist = 74954), *TNFSF8* (dist = 12261)	0 (0)	11 (6)	2 (0)	NA	2	Whole Blood (*TNFSF8*)
		rs7028891^e^	117645015	intergenic	*TNFSF15* (dist = 76607), *TNFSF8* (dist = 10608)	0 (0)	4 (3)	1 (1)	NA	3	Whole Blood (*TNFSF8*)

Abbreviations: EID, epigenome identifier; dist, distance; #, number; DHS, DNase I hypersensitivity site; eQTL, expression quantitative trait loci.

^a^Counts of the number of consolidated cell types (EIDs) for which the SNP of interest overlaps the queried epigenomic assay peak (Roadmap Epigenomics Mapping Consortium processed data, Kundaje *et al*.)^21^. “PBC EID”: Separately considers peak overlap counts among the 29 blood/liver cell types available in Roadmap Epigenomics.

^b^Bound protein: Regulatory protein-binding ChIP-seq peak overlaps for specified proteins are provided for blood- or liver-related cell lines only (HaploReg v4, Ward and Kellis)^37^.

^c^Altered motifs: The number of regulatory motifs predicted to be affected by the SNP based on position weight matrices (PWM) score changes (HaploReg v4, Ward and Kellis)^37^.

^d^eQTLs: Reported significant eQTLs for whole blood, lymphoblastoid, and liver cell types only (GTEx Consortium^20^; HaploReg v4, Ward and Kellis)^37^.

^e^Signifies non-HLA SNPs.

## Discussion

In the current study, we propose a novel approach for the detection of haplotype associations to detect PBC risk haplotypes genome-wide. Previous studies have successfully combined agnostic sliding windows with an exhaustive testing strategy to identify haplotypes associated with disease risk. To contrast, our proposed method uses a logic regression-based stochastic search to detect best 3-SNP haplotype associations among phased SNP alleles mapped to broadly-defined gene regions. This approach has several strengths. First, the chosen analytic windows encourage searches for genic haplotypes, thereby finding combinations of variably-spaced SNPs that may influence *cis*-regulatory mechanisms for gene expression. Second, logic regression considers multiple models of risk (e.g., presence of risk alleles at *either* of two loci) that can better reflect regulatory redundancies that may exist in controlling transcription. Lastly, the proposed method avoids exhaustive testing, potentially capturing true haplotype associations that would otherwise be missed due to lack of statistical power. Using the proposed method, a total of 74 3-SNP haplotypes on chromosomes 6, 7, and 9 were considered as having stronger associations with PBC risk than expected under a permutation-based approach in a discovery cohort of 1,937 Japanese individuals. Nearly two-thirds of these selected haplotypes (49 haplotypes) were replicated in a second independent Japanese cohort (N = 949) after applying a Bonferroni-corrected p-value threshold (P < 6.8 × 10^−4^).

Haplotype association analyses using inferred haplotypes in downstream analyses may be vulnerable to Type I error inflation and biased estimates of genetic effects due to misclassification of haplotype states^25^. Several aspects of this analysis mitigate these concerns. Phasing was conducted for cases and controls simultaneously, which provides greater control of Type I error than phasing these groups separately^25^. Differential misclassification of haplotype states is unlikely, since haplotype phasing was conducted without knowledge of disease status. Non-differential misclassification is more plausible; thus, reported effect estimates may be biased towards the null (haplotype has no effect on PBC risk). To safeguard against false positives, we conducted a replication study and applied a Bonferroni-corrected p-value threshold to address multiple testing in replication. Our benchmark method analysis that considered the uncertainty of haplotype phase also tagged ~82% of the gene analytic windows containing replicated 3-SNP haplotypes. Lastly, the frequency distributions for replicated 3-SNP haplotypes among controls in our study were consistent with the 1000G JPT reference panel. These results suggest that our method can reliably detect credible 3-SNP genic haplotypes associated with PBC risk.

Limitations of our proposed method include not finding the haplotype with the strongest association (due to non-exhaustive testing) and missing risk haplotypes outside of genic regions. However, we replicated 49 out of 74 genic haplotype associations selected in discovery; replicated 3-SNP haplotypes also frequently included SNPs that overlapped top haplotype associations identified with the exhaustive testing-based benchmark method. Yet, exact matches between top haplotypes detected with the proposed and benchmark methods were not observed. Instead, replicated 3-SNP haplotypes detected by logic regression linked SNPs ~335 kb apart on average, with many contributing SNPs overlapping functional annotations in cell/tissue types relevant to PBC. Thus, the haplotypes detected with the proposed method: (a) frequently include SNPs that overlap genomic regions with top haplotype associations identified by an exhaustive testing-based benchmark method; (b) are unlikely to be detected with existing association methods; and (c) combine the effects of variably-spaced and potentially functional SNPs mapped to regions of the genome that are more likely to be transcribed.

Similar to recent PBC GWAS^4,5,8^, we did not examine sex chromosome variants. However, sex chromosome-related defects and haplotype deficiencies likely play an important role in PBC etiology^26,27^. Specifically, higher rates of X monosomy in peripheral blood cells in PBC-affected women have been observed, suggesting X monosomy influences PBC pathogenesis^28^. Although skewed X-chromosome inactivation (XCI) may contribute to autoimmune disease risk^29,30^, preferential X loss that involves particular X-linked haplotypes may better explain the increased X monosomy in women with PBC^31^. Analogous to this X haploinsufficiency observed in women with PBC, increased Y chromosome loss has been associated with PBC in men^32^. Further exploration of sex chromosome-specific haplotype associations with PBC risk is needed.

While we did not functionally validate replicated 3-SNP haplotypes, results from ancillary bioinformatics analyses suggest that identified haplotypes may be considered in future functional investigations of genetic susceptibility factors for PBC. SNPs in replicated 3-SNP haplotypes were significantly enriched for gene expressions in lymphoblastoid cells and indicators of enhancer, promoter, and open chromatin states in blood and liver cell types compared to SNPs in extended genic regions that were marginally associated with PBC risk. Specific functional annotations of SNPs in PBC-associated haplotypes also indicate that identified haplotypes may enhance understanding of posited disease mechanisms for both novel and known genetic associations. For example, the chromosome 7 haplotype, ((rs12671658 = T) or (rs12702656 = A)) and (rs11768586 = G), represents a novel candidate PBC susceptibility locus, with SNPs mapped to intronic regions of *UMAD1*. Two SNPs in this haplotype (rs12702656, rs11768586) overlap enhancer peaks in liver cells. A suggestive association with insulin-like growth factor 1 (IGF1) levels, a hormone predominantly produced by the liver, has been reported for a proximal variant mapped to *UMAD1* (rs7780564, p = 3.9 × 10^−7^)^33^; IGF1 is hypothesized to regulate biliary epithelial cell proliferation^34^. On the other hand, *TNFSF15* is reported to be the most strongly associated non-HLA genetic susceptibility factor for PBC in Japanese. *TNFSF15* is anticipated to play a role in the inflammation response, activating Th1/Th17 cell differentiation and cytokine production^9^. The (rs4979484 = C) or ((rs13300483 = T) and (rs7028891 = G)) haplotype, comprised of intergenic SNPs near *TNFSF15*, implicate *TNFSF8* as another possible contributor to PBC risk. This 3-SNP haplotype includes two SNPs significantly associated with *TNFSF8* expression and a third SNP (rs4979484) with evidence of NF-kB binding in GM18951 (JPT) lymphoblastoid cells. *TNFSF8* specifically encodes a cytokine (CD30L) expressed in T cells and monocytes; like *TNFSF15*, *TNFSF8* is also a known activator of NF-kB, a transcription factor implicated in inflammation and immunity pathways^24^.

In conclusion, this study presents a novel approach to conduct haplotype association analyses genome-wide, with a deliberate focus on interrogating regions of the genome that are likely to be transcribed. With this method, we identified novel candidate PBC susceptibility loci (e.g., *UMAD1*) and detected haplotype patterns that potentially contribute to hypothesized disease pathways that include previously reported PBC susceptibility genes (*HLA-DRA*, *TNFSF15*) in the Japanese population. Broader explorations of haplotypes may increase understanding of the genetic basis of PBC and potentially inform new interventions that improve disease prognosis.

## Methods

### Study population

The current study combines data collected for two previous PBC GWAS in Japanese, both coordinated by the Japan PBC-GWAS Consortium^8,9^. All research participants provided informed consent. The methods/protocols implemented in this analysis were approved by the ethics committees of the Nagasaki Medical Center and all participating institutions. All methods were performed in accordance with relevant guidelines and regulations.

The combined study population has been previously described in detail^8^. Briefly, DNA samples were obtained from either healthy controls reporting no apparent disease or PBC cases, defined by laboratory or histological evidence of at least two of the following criteria: cholestasis, ascertained by elevated alkaline phosphatase; serum anti-mitochondrial antibodies; and non-suppurative destructive cholangitis and interlobular bile duct destruction. DNA samples were genotyped using the Affymetrix Axiom Genome-Wide ASI 1 Array (Affymetrix, Santa Clara, CA). As previously reported^8^, samples with excess heterozygosity rates, cryptic relatedness, or of non-Japanese ancestry were removed. A total of 425,290 autosomal SNPs were retained under the following quality control criteria: SNP call rate ≥95%; MAF ≥5%; and Hardy-Weinberg equilibrium (HWE) P ≥ 0.001 in controls. Samples with <97% sample call rate among retained SNPs were excluded, resulting in a study sample of 2,886 individuals. We split this sample *a priori* into two cohorts to correspond with the timing of sample collections for the two previous PBC GWAS: 1,937 participants (901 cases, 1,036 controls) for haplotype signal discovery, and 949 participants (480 cases, 469 controls) for haplotype signal replication.

### Haplotype phasing

Haplotype phase was computationally estimated using SHAPEIT v2.79^35^ for whole chromosomes with unphased SNP genotypes for all 2,886 unrelated samples in the combined study cohort. To improve phasing accuracy, we used 1000 Genomes Phase 3 genetic map recombination rates, increased the number of conditioning states for the haplotype estimation to 600 states, and increased the numbers of burn-in (10), pruning (10), and main iterations (50) of the SHAPEIT MCMC algorithm. Recommended parameters for haplotype estimation mean window size (2 Mb) and effective population size (15,000) for GWAS data were employed. Upon phasing, each sample was assigned its most likely haplotype phase configuration with binary-encoded phased genotypes at each SNP locus (0|0, 0|1, 1|0, or 1|1, where 0 = reference allele, 1 = alternative allele).

### Statistical Analyses

#### Restricting haplotype formation within gene regions

We restricted our search for haplotype signals within regions centered on annotated protein-coding and non-translated RNA-encoding genes. Specifically, we used gene transcripts annotated by the RefSeq gene model (release 74, GRCh37/hg19 build)^36^, employed ANNOVAR^37^ to map SNPs in our dataset to introns, exons, and 3′/5′ untranslated regions, and identified flanking 500-kb regions for each RefSeq transcript, as flanking gene regions are critical for transcriptional events^38^. SNPs mapped to a gene and flanking regions were considered as a single “gene analytic window” (Supplementary Fig. S1). To reduce each set of SNPs in a given gene analytic window to “tagging SNPs” in formulating haplotypes, we removed SNPs within each window sequentially so that no pair of SNPs was in high linkage disequilibrium (r^2^ ≤ 0.8). Gene analytic windows with a minimum of two SNPs were retained for analysis.

#### Detecting 3-SNP haplotypes with logic regression

We identified gene-based haplotype patterns consisting of three SNPs associated with PBC risk with an adapted logic regression^18^ algorithm. Briefly, logic regression is a statistical learning method that utilizes a stochastic search algorithm to support the detection of higher order interactions associated with an outcome within a generalized linear model (GLM) framework. For our analysis, 3-SNP haplotypes are expressed mathematically as Boolean (true/false) variables that combine SNP alleles on the same chromosome, e.g., ((SNP1 = reference allele) and (SNP2 = alternative allele) and (SNP3 = alternative allele)) = {True, False}.

We utilized logic regression’s “simulated annealing” search algorithm (R “LogicReg” package, version 1.5.8) to build many possible haplotype predictors stochastically and evaluate them based on GLM model scores (e.g., deviance scores for logistic regression). Logic regression was applied to each gene analytic window, considering the contributions of two haplotypes per subject in our discovery cohort (N = 1,937), to identify candidate 3-SNP haplotype patterns associated with PBC risk. To stabilize the performance of the stochastic algorithm, we utilized 100 different initialization values for each gene analytic window and chose the model with the lowest deviance score among the 100 fits.

To select candidate 3-SNP haplotypes for replication, we used a permutation-based evaluation statistic based on previous work^19^. Specifically, for each gene analytic window, 20 permutations of PBC disease status were used to obtain model deviance scores under each permutation. We then calculated the 20 possible null distribution deviations between a deviance score under a given permutation and the median estimated with the remaining 19 permuted datasets, along with the corresponding median absolute deviation (MAD, a robust measure of variability) for each gene window’s set of 20 null distribution deviations. We defined our permutation-based evaluation statistic as $\frac{{D}_{obs}-{D}_{med}}{MA{D}_{{D}_{med}}}$, where *D*_*obs*_ is the observed deviance score for the gene analytic window’s best-fitting 3-SNP haplotype, *D*_*med*_ is its respective empirically-derived median under 19 permutations, and $MA{D}_{{D}_{med}}$ is the MAD of the null distribution deviations under 20 permutations.

Given gene analytic windows potentially overlap, the same best-fitting 3-SNP haplotype could be identified for multiple windows. In these cases, the 3-SNP haplotype with the best permutation-based evaluation statistic across multiple windows was retained to contribute to a set of unique 3-SNP haplotypes for replication follow-up (e.g., no haplotype tree had an exact SNP and logic tree structure match with another tree). We set an *a priori* threshold to select candidate 3-SNP haplotypes: 3-SNP haplotypes with the top 1% of permutation-based evaluation statistics among the set of unique 3-SNP haplotypes were selected for replication, translating to statistic values of less than −11.4. This cut-off translates to the selection of 3-SNP haplotypes with logistic regression model deviance scores at least 11.4 median absolute deviations away from corresponding medians estimated under the null distribution.

Since haplotype logic regression models consider two observations with the same case/control status from each subject (i.e., two haplotypes from two homologous chromosomes), we used the following logistic regression model for the best detected 3-SNP haplotype associations, treating each subject as an independent observation to satisfy GLM assumptions for valid statistical inference:

1 $$\mathrm{log}(\frac{p}{1-p})={\beta }_{0}+{\beta }_{1}L$$

where *p* is PBC risk and *L* represents additive haplotype effects of the best-fitting 3-SNP haplotype logic tree. The reported model deviance score, odds ratio, and p-value for each detected haplotype in the discovery cohort were derived from equation 1.

To replicate selected haplotype associations, we tested the selected 3-SNP haplotypes from the discovery cohort with the same model described in equation 1 in our independent replication cohort (N = 949) and applied a Bonferroni-corrected per-test significance threshold (P < 0.05/number of selected haplotypes). We further required odds ratios in the replication cohort to have the same direction as the discovery cohort to consider a 3-SNP haplotype as replicated.

#### Detecting 3-SNP haplotypes with a benchmark method

We used the R “haplo.stats” package^14^, commonly employed in secondary explorations of haplotypes in single-SNP GWAS^39,40^, as our benchmark haplotype association analysis method. This method is used to obtain maximum likelihood estimates for haplotype frequencies using the expectation-maximization (EM) algorithm. Associations between haplotypes and disease risk are tested with a score statistic for a global test of haplotypes (*h*-1 degrees of freedom for *h* haplotypes, treating the most common haplotype as a reference), with variance estimates that account for the uncertainty in the haplotype estimation. We used this method to exhaustively test 3-SNP haplotypes consisting of contiguous SNPs (with estimated haplotype counts of at least 20), using a 3-SNP sliding window strategy with a skip length of one SNP, among SNPs in gene analytic windows.

#### Ancillary bioinformatics analyses

We performed functional annotations and enrichment analyses with HaploReg v4^41^, Roadmap Epigenomics Mapping Consortium (REMC)^21^, and Genotype-Tissue Expression (GTEx Analysis v7)^20^ resources for SNPs in replicated 3-SNP haplotypes. We conducted enrichment analyses using REMC histone modification ChIP-seq peak (gappedPeak algorithm) data for H3K4me3 (promoter) and H3K4me1 (enhancer) marks, and DNase I hypersensitivity peak (narrowPeak algorithm) data for all available consolidated blood/liver human cell types (29 and 11 cell types available, respectively). For each cell type, we compared the set of SNPs in replicated haplotypes with a set of 16,036 non-overlapping SNPs mapped to gene analytic windows, each with nominal single-SNP associations (P < 0.05) with PBC risk. Frequencies of overlap between SNPs in each set and epigenomic peaks were counted in each cell type. We evaluated enrichment for each assay using Bonferroni-corrected p-values obtained from 2-sided Fisher’s exact tests. To investigate enrichments in gene expressions for SNPs in replicated haplotypes among the three blood- and liver-related tissue types available in GTEx, counts of significant *cis*-eQTLs (SNPs within +/−1 Mb of transcription start sites, q-value < 0.05) for haplotype SNPs were compared to the aforementioned comparison SNP set using a 2-sided Fisher’s exact test. Visualizations highlighting functional annotations of selected 3-SNP haplotypes were created with the “Gviz” R/Bioconductor package^42^.

### Data availability statement

The genetic data analyzed in this study are available in the European Genome-phenome Archive under study accession number EGAS00001002915 (https://www.ebi.ac.uk/ega/studies/EGAS00001002915). All other analyzed data are included in this published article or the Supplementary Information file.

## Electronic supplementary material

Supplementary Information
