# Supplementary material for: Genome-wide haplotype association analysis of primary biliary cholangitis risk in Japanese
Source: Sci Rep. 2018 May 17;8:7806. doi: 10.1038/s41598-018-26112-1 (PMC5958065; doi:10.1038/s41598-018-26112-1)
Supplement: Supplementary file 1 — Supplementary Information [file 41598_2018_26112_MOESM1_ESM.pdf]

Supplementary Information

Title: Genome-wide haplotype association analysis of primary biliary cholangitis risk in Japanese

Authors: Cindy Im,[1]* Yadav Sapkota,[2] Wonjong Moon,[2] Minae Kawashima,[3] Minoru Nakamura,[4] Katsushi Tokunaga,[3] Yutaka Yasui[1,2]*

Affiliations:
1. School of Public Health, University of Alberta, Edmonton, Alberta T6G 1C9, Canada
2. Department of Epidemiology and Cancer Control, St. Jude Children's Research Hospital, Memphis, TN 38105, USA
3. Department of Human Genetics, Graduate School of Medicine, The University of Tokyo, Tokyo 113-0033, Japan
4. Department of Hepatology, Nagasaki University Graduate School of Biomedical Sciences and Clinical Research Center, National Hospital Organization Nagasaki Medical Center, Omura, Nagasaki, 856-8562, Japan

Supplementary Figure S1: Example schematic of RefSeq gene-based analytic windows used for haplotype formation in the main analysis. The bracketed region highlighted in red, which includes the RefSeq gene transcript and flanking 500-kb regions before and after transcription start/stop sites, spans typical genomic elements included in any given gene analytic window.

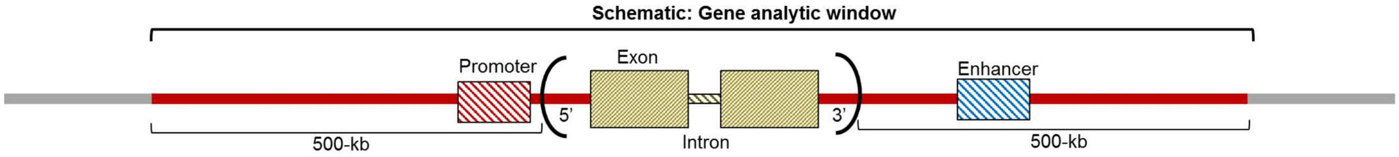

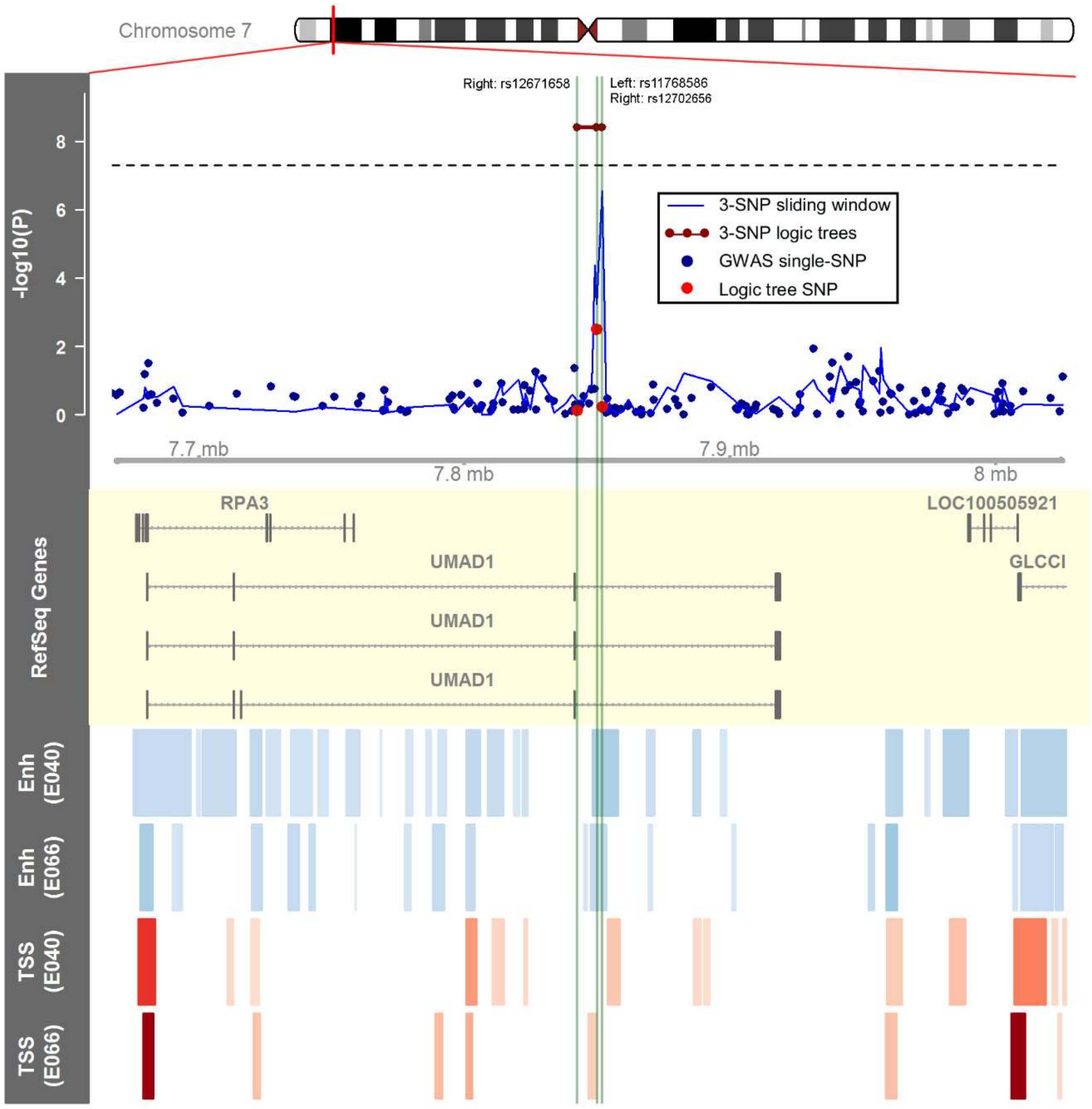

Supplementary Figure S2: Visualization of the region surrounding the replicated 3-SNP haplotype logic tree for chromosome 7 (chr7:7667281-8026742, hg19; corresponds to the red band in the chromosomal ideogram). The top data track shows single-SNP association results and haplotype association results using benchmark ("3-SNP sliding window") and proposed ("3-SNP logic tree") methods for the selected genomic region. The annotation tracks beneath show RefSeq genes, a heatmap corresponding to Roadmap Epigenomics[21] H3K4me1 (Enh or enhancer) and H3K4me3 (TSS or promoter) ChIP-seq peaks in primary T helper memory cells (E040) and liver cells (E066). No significant eQTLs were associated with SNPs in replicated haplotypes.

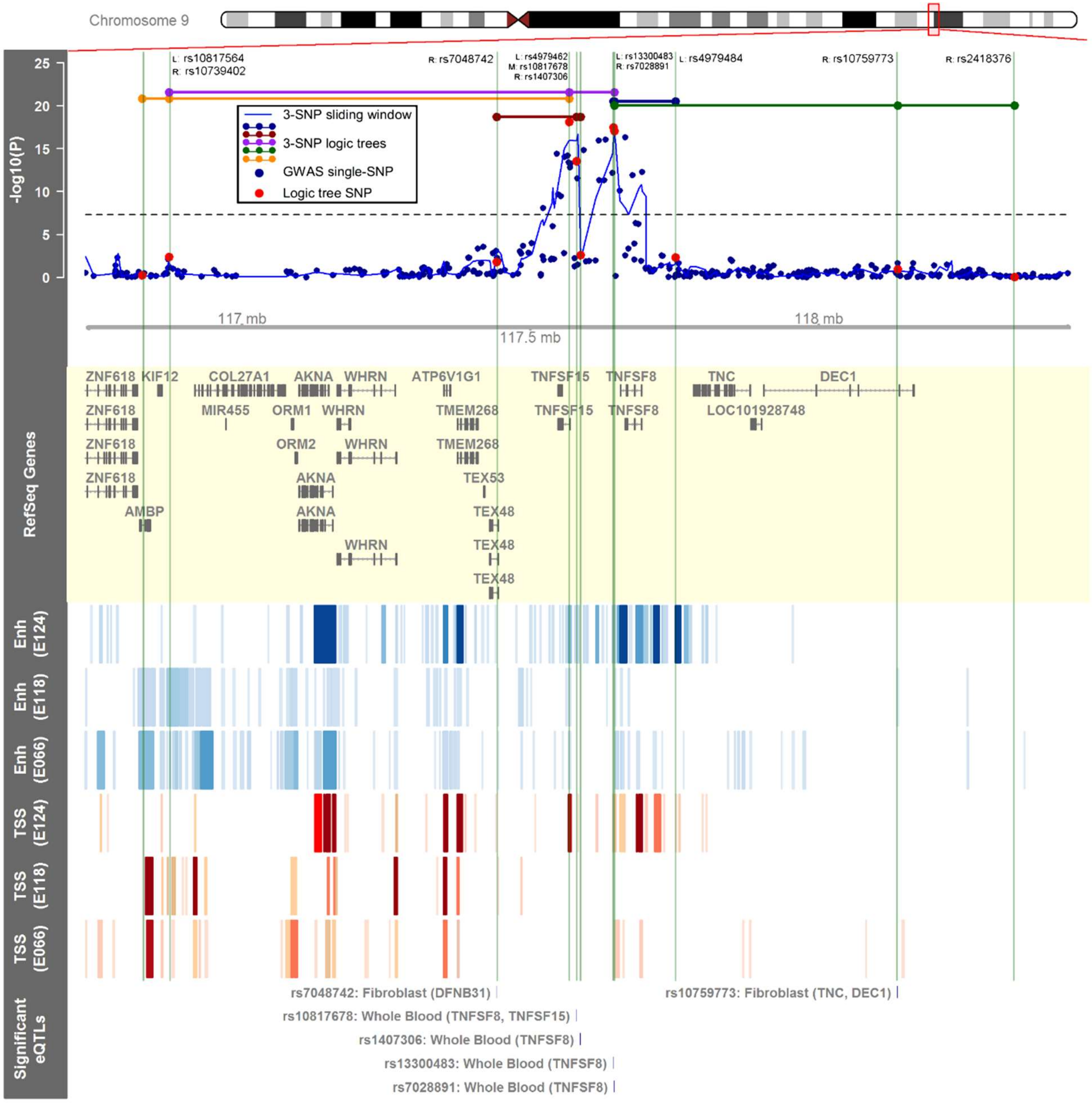

Supplementary Figure S3: Visualization of the region surrounding the five replicated 3-SNP haplotype logic trees in chromosome 9 (chr9:116727079-118438852, hg19; corresponds to the red rectangle in the chromosomal ideogram). The top data track shows single-SNP association results and haplotype association results using benchmark (“3-SNP sliding window”) and proposed (“3-SNP logic tree”) methods for the selected genomic region. The annotation tracks beneath show RefSeq genes, a heatmap corresponding to Roadmap Epigenomics[21] H3K4me1 (Enh or enhancer) and H3K4me3 (TSS or promoter) ChIP-seq peaks in monocytes (E124), hepatocellular carcinoma cells or HepG2 (E118), and liver cells (E066), and all significant eQTLs associated with SNPs in replicated haplotypes in blood, liver, and fibroblast cell types (GTEx Consortium[20]).

Supplementary Table S1: Summary of discovery and replication analysis results for the 49 replicated 3-SNP haplotypes

				DISCOVERY (N=1,937)			REPLICATION (N=949)		COMBINED (N=2,886)				
Chr	3-SNP haplotype tree	RefSeq gene windows with 3-SNP haplotype	# SNPs in gene windows	Permutation-based selection statistic	OR	P	OR	P	OR	P	# with 0 haplotype copies (% cases)	# with 1 haplotype copy (% cases)	# with 2 haplotype copies (% cases)
6	(rs3117106=C) and ((rs206018=G) or (rs9501179=A))	*SLC44A4; EHMT2*	148-158	-82.148	0.169	3.3×10^{-17}	0.257	2.7×10^{-7}	0.196	3.0×10^{-23}	2,609 (51.2%)	264 (17.4%)	13 (0.0%)
6	(rs3129881=C) or ((rs375244=A) and (rs3132947=G))	*CFB; NELFE; C2; C2-AS1*	153-163	-69.730	4.937	1.8×10^{-20}	2.324	1.6×10^{-5}	3.665	2.3×10^{-24}	17 (17.6%)	360 (21.7%)	2,509 (51.8%)
6	((rs35372932=T) and (rs9269190=C)) or (rs9270493=C)	*TNXB*	157	-58.438	0.325	4.3×10^{-25}	0.451	1.7×10^{-8}	0.365	9.6×10^{-32}	2,162 (54.3%)	656 (29.7%)	68 (16.2%)
6	(rs9268977=T) or ((rs3135395=T) or (rs550513=T))	*SKIV2L*	159	-57.402	3.741	1.7×10^{-21}	2.991	1.0×10^{-9}	3.448	1.2×10^{-29}	43 (16.3%)	439 (23.0%)	2,404 (53.0%)
6	((rs9268634=G) or (rs35344500=C)) and (rs9275175=G)	*NOTCH4; GPSM3*	177-193	-47.911	0.417	6.1×10^{-27}	0.500	6.8×10^{-11}	0.448	1.3×10^{-35}	1,606 (57.8%)	1,069 (38.2%)	211 (21.3%)
6	((rs9268831=T) or (rs9269190=T)) or (rs9270652=C)	*ATF6B; FKBPL; PPT2; PPT2-EGFL8; AGPAT1*	158-169	-43.565	3.640	3.2×10^{-23}	2.315	1.9×10^{-7}	3.075	7.3×10^{-29}	46 (15.2%)	496 (26.0%)	2,344 (53.1%)
6	(rs3132947=G) or ((rs9501179=G) and (rs41546114=C))	*C6orf48; SNORD48*	162	-42.196	6.316	1.2×10^{-16}	3.533	7.9×10^{-7}	5.030	2.4×10^{-22}	11 (0.0%)	257 (17.5%)	2,618 (51.0%)
6	(rs9268831=T) or ((rs2395194=G) or (rs387608=A))	*STK19*	160	-38.720	3.869	8.0×10^{-22}	3.169	4.3×10^{-10}	3.601	2.2×10^{-30}	42 (16.7%)	431 (22.0%)	2,413 (53.0%)
6	(rs3132947=G) or ((rs9268014=C) and (rs41546114=C))	*MSH5*	181	-38.294	6.221	5.3×10^{-17}	3.533	7.9×10^{-7}	5.012	1.0×10^{-22}	12 (0.0%)	261 (17.6%)	2,613 (51.1%)
6	(rs3132947=G) or ((rs9268055=T) and (rs41546114=C))	*MSH5-SAPCD1*	181	-38.294	6.221	5.3×10^{-17}	3.533	7.9×10^{-7}	5.012	1.0×10^{-22}	12 (0.0%)	261 (17.6%)	2,613 (51.1%)
6	((rs241437=G) or (rs3129299=C)) or (rs9276909=C)	*PFDN6; WDR46; VPS52; DAXX; RPS18; HCG25*	125-139	-37.551	6.468	5.0×10^{-16}	2.912	2.2×10^{-5}	4.716	1.8×10^{-20}	16 (0.0%)	237 (18.6%)	2,633 (50.8%)
6	((rs2395194=G) and (rs16870908=G)) or (rs9268831=T)	*HLA-DMA; HLA-DQB2; LOC100294145; C6orf10; HLA-DOB; TAP2; HLA-DQA2; HCG23; HLA-DMB; BTNL2; PSMB9; TAP1; HLA-DQA1; HLA-DQB1; HLA-DRA; HLA-DRB6*	197-283	-35.650	2.995	1.7×10^{-25}	2.292	4.4×10^{-9}	2.732	5.9×10^{-33}	72 (11.1%)	690 (31.2%)	2,124 (54.5%)
6	((rs9268213=A) and (rs41546114=C)) or (rs3132947=G)	*HSPA1L*	158	-35.026	6.221	5.3×10^{-17}	3.533	7.9×10^{-7}	5.012	1.0×10^{-22}	12 (0.0%)	261 (17.6%)	2,613 (51.1%)
6	((rs4538748=C) or (rs2064476=G)) and (rs35344500=A)	*HLA-DOA; BRD2; HLA-DPB2; HLA-DPA1; HLA-DPB1*	187-192	-34.656	2.406	9.3×10^{-26}	2.136	5.4×10^{-11}	2.313	3.3×10^{-35}	173 (20.8%)	941 (35.8%)	1,772 (56.9%)
6	((rs2244027=A) or (rs2242665=C)) or (rs2071591=G)	*MICB; LY6G5B; PRRC2A; ATP6V1G2-DDX39B; DDX39B; MCCD1; NFKBIL1; AIF1; ATP6V1G2; DDX39B-AS1; LST1; LTA; APOM; CSNK2B; GPANK1; BAG6; HCP5; MICA*	152-203	-27.403	2.943	3.4×10^{-19}	2.659	9.3×10^{-8}	2.873	8.7×10^{-26}	33 (21.2%)	509 (26.7%)	2,344 (52.8%)
6	((rs9296088=A) and (rs12207818=C)) and (rs181997=A)	*KIFC1; SYNGAP1*	118-123	-26.700	0.142	1.3×10^{-14}	0.377	6.1×10^{-4}	0.204	6.6×10^{-18}	2,671 (50.4%)	205 (17.6%)	10 (0.0%)
6	((rs2071287=C) and (rs3094596=C)) or (rs185819=C)	*ABHD16A*	152	-22.999	3.720	6.5×10^{-18}	2.882	3.2×10^{-8}	3.370	1.5×10^{-24}	29 (3.4%)	379 (25.1%)	2,478 (51.9%)
6	(rs3132947=T) and ((rs404860=C) or (rs41546114=T))	*C6orf25*	169	-21.846	0.186	8.2×10^{-17}	0.382	7.7×10^{-6}	0.253	3.2×10^{-21}	2,584 (51.0%)	288 (21.5%)	14 (0.0%)
6	(rs11754586=T) or ((rs3131932=G) or (rs9262537=G))	*PPP1R10; ABCF1; ATAT1*	138-148	-21.366	3.889	1.0×10^{-15}	2.396	2.6×10^{-5}	3.241	1.8×10^{-19}	13 (7.7%)	327 (24.5%)	2,546 (51.1%)

				DISCOVERY (N=1,937)			REPLICATION (N=949)		COMBINED (N=2,886)				
Chr	3-SNP haplotype tree	RefSeq gene windows with 3-SNP haplotype	# SNPs in gene windows	Permutation-based selection statistic	OR	P	OR	P	OR	P	# with 0 haplotype copies (% cases)	# with 1 haplotype copy (% cases)	# with 2 haplotype copies (% cases)
6	((rs10947251=A) or (rs41546114=T)) and (rs3132947=T)	*VARS*	173	-20.943	0.166	1.8×10^{-16}	0.278	5.4×10^{-7}	0.203	2.8×10^{-22}	2,616 (51.0%)	258 (17.8%)	12 (0.0%)
6	((rs3132947=G) or (rs9266632=G)) or (rs185819=C)	*LY6G6E*	154	-20.783	5.008	3.8×10^{-17}	3.426	1.0×10^{-7}	4.321	2.0×10^{-23}	14 (0.0%)	298 (20.1%)	2,574 (51.3%)
6	(rs805273=A) and ((rs805267=G) or (rs9266774=T))	*HCG27*	222	-20.015	0.229	8.2×10^{-17}	0.337	7.2×10^{-7}	0.264	3.1×10^{-22}	2,555 (51.3%)	317 (22.4%)	14 (0.0%)
6	(rs3828901=A) or ((rs707921=A) and (rs9266774=T))	*CCHCR1; TCF19*	222	-19.687	0.231	6.9×10^{-17}	0.382	6.0×10^{-6}	0.279	2.5×10^{-21}	2,551 (51.2%)	322 (23.3%)	13 (0.0%)
6	(rs3828901=G) and ((rs805268=A) or (rs9266774=C))	*POU5F1*	222	-19.444	4.354	1.1×10^{-16}	2.618	6.9×10^{-6}	3.586	4.4×10^{-21}	12 (0.0%)	319 (23.2%)	2,555 (51.2%)
6	(rs3828901=A) or ((rs3828919=A) and (rs2523497=T))	*MUC22*	219	-19.050	0.231	4.2×10^{-17}	0.479	1.6×10^{-4}	0.313	9.0×10^{-20}	2,531 (51.1%)	345 (25.2%)	10 (0.0%)
6	(rs707922=G) or ((rs1046089=A) and (rs9266774=C))	*PSORS1C3*	222	-17.984	4.314	1.6×10^{-16}	2.967	7.2×10^{-7}	3.748	6.2×10^{-22}	14 (0.0%)	315 (22.5%)	2,557 (51.2%)
6	((rs3131932=G) or (rs28360042=C)) or (rs885950=C)	*NRM; MDC1; MDC1-AS1; DHX16; PPP1R18; TUBB; FLOT1; IER3; DPCR1; DDR1; GTF2H4; LINC00243*	146-211	-17.890	3.907	7.4×10^{-16}	2.650	2.3×10^{-6}	3.356	1.1×10^{-20}	14 (0.0%)	334 (24.6%)	2,538 (51.2%)
6	((rs9266774=T) and (rs2255741=A)) or (rs3828901=A)	*PSORS1C2*	221	-17.684	0.234	1.4×10^{-16}	0.371	3.4×10^{-6}	0.279	2.6×10^{-21}	2,551 (51.2%)	323 (23.2%)	12 (0.0%)
6	(rs3749946=A) and ((rs2517506=T) and (rs7741091=A))	*MUC21*	209	-17.342	0.234	3.1×10^{-16}	0.394	9.8×10^{-6}	0.287	1.9×10^{-20}	2,554 (51.1%)	322 (23.6%)	10 (0.0%)
6	(rs3828901=G) and ((rs12110785=T) or (rs2523644=T))	*PSORS1C1; HCG22*	218-223	-15.815	4.572	2.9×10^{-16}	2.427	3.0×10^{-5}	3.544	6.3×10^{-20}	10 (0.0%)	306 (23.2%)	2,570 (51.0%)
6	((rs2239888=T) or (rs3134769=C)) and (rs3828901=G)	*CDSN*	221	-14.656	4.240	1.2×10^{-16}	2.578	7.7×10^{-6}	3.510	5.5×10^{-21}	11 (0.0%)	327 (23.5%)	2,548 (51.2%)
6	(rs2517681=T) or ((rs4148248=C) and (rs2735078=A))	*TRIM10; TRIM26; TRIM31; TRIM31-AS1; TRIM40; TRIM15*	93-96	-13.047	3.254	5.1×10^{-13}	3.065	1.1×10^{-6}	3.196	2.6×10^{-18}	13 (7.7%)	309 (24.6%)	2,564 (50.9%)
6	(rs17195733=G) or ((rs13201129=C) and (rs1737069=T))	*HLA-L*	93	-13.034	0.361	2.0×10^{-14}	0.364	2.2×10^{-7}	0.360	1.8×10^{-20}	2,444 (51.7%)	419 (27.2%)	23 (17.4%)
6	(rs3130785=T) and ((rs9261301=G) or (rs1264570=C))	*RPP21*	100	-12.876	0.291	6.0×10^{-14}	0.441	6.7×10^{-5}	0.340	2.7×10^{-17}	2,549 (50.8%)	323 (25.7%)	14 (14.3%)
6	(rs2517681=C) and ((rs3130785=T) or (rs2735078=G))	*TRIM39; TRIM39-RPP21*	103	-11.587	0.289	4.3×10^{-14}	0.376	5.4×10^{-6}	0.317	1.2×10^{-18}	2,552 (51.0%)	320 (24.4%)	14 (14.3%)
6	((rs1345229=A) or (rs1233387=T)) or (rs1003581=G)	*HCG4; LOC554223; HLA-F; HLA-G*	113-117	-11.537	3.168	6.9×10^{-13}	2.885	1.7×10^{-6}	3.070	5.4×10^{-18}	14 (7.1%)	318 (25.5%)	2,554 (50.9%)
6	((rs4713429=G) or (rs12110785=T)) or (rs3131932=G)	*GNL1; PRR3*	139	-11.517	3.661	8.4×10^{-16}	2.344	2.0×10^{-5}	3.093	1.0×10^{-19}	17 (0.0%)	346 (26.3%)	2,523 (51.1%)
6	((rs1003581=G) and (rs16894681=T)) or (rs1233387=T)[a]	*OR2H2; UBD; OR10C1; OR11A1; OR12D2; OR12D3; OR5V1; OR2H1; GABBR1; OR2J2; OR2J3*	78-110	-14.375	2.697	6.4×10^{-13}	2.255	1.9×10^{-5}	2.541	5.5×10^{-17}	18 (5.6%)	401 (29.9%)	2,467 (51.1%)
6	((rs2281043=T) or (rs7751451=G)) and (rs1635=A)[a]	*ZSCAN12; ZKSCAN3; PGBD1; ZSCAN31; ZSCAN23; ZBED9; GPX5; GPX6*	50-53	-14.306	0.377	2.7×10^{-11}	0.360	1.7×10^{-6}	0.371	2.1×10^{-16}	2,527 (50.8%)	341 (27.9%)	18 (11.1%)

				DISCOVERY (N=1,937)			REPLICATION (N=949)		COMBINED (N=2,886)				
Chr	3-SNP haplotype tree	RefSeq gene windows with 3-SNP haplotype	# SNPs in gene windows	Permutation-based selection statistic	OR	P	OR	P	OR	P	# with 0 haplotype copies (% cases)	# with 1 haplotype copy (% cases)	# with 2 haplotype copies (% cases)
6	((rs3117192=C) or (rs16894216=T)) and (rs7773193=T)[a]	*ZNF311; LINC01556; OR2W1*	61-67	-14.047	2.709	2.6×10^{-11}	2.728	1.5×10^{-6}	2.711	2.0×10^{-16}	17 (11.8%)	340 (27.6%)	2,529 (50.8%)
6	(rs9295704=C) and ((rs2451752=A) and (rs2575174=C))[a]	*BTN3A2; BTN2A2*	46-52	-12.263	0.362	1.7×10^{-10}	0.374	1.3×10^{-5}	0.365	8.9×10^{-15}	2,571 (50.4%)	304 (27.3%)	11 (9.1%)
6	(rs6939576=G) or ((rs2859365=G) or (rs6930033=A))[a]	*TRIM27*	64	-12.215	2.681	9.9×10^{-11}	3.120	3.3×10^{-7}	2.820	1.7×10^{-16}	20 (15.0%)	312 (26.3%)	2,554 (50.7%)
6	(rs7773193=C) or ((rs17280818=T) and (rs2394100=T))[a]	*NKAPL; ZSCAN26; ZKSCAN4; ZSCAN9*	52-53	-11.424	0.383	4.3×10^{-11}	0.383	3.2×10^{-6}	0.382	6.3×10^{-16}	2,524 (50.8%)	342 (28.7%)	20 (10.0%)
7	((rs12671658=T) or (rs12702656=A)) and (rs11768586=G)[a]	*GLCCI1; LOC100505921; ICA1; COL28A1; MIOS; RPA3; LOC100505938; UMAD1; LOC101927391*	204-292	-16.424	0.040	8.7×10^{-6}	0.112	3.6×10^{-4}	0.066	3.9×10^{-9}	2,802 (49.1%)	84 (6.0%)	0 (NA)
9	(rs4979484=C) or ((rs13300483=T) and (rs7028891=G))[a]	*LOC100505478; TNFSF15; C9orf91; TNFSF8*	121-156	-28.431	1.746	4.2×10^{-17}	1.528	8.6×10^{-6}	1.672	3.0×10^{-21}	775 (35.4%)	1,434 (48.7%)	677 (60.4%)
9	(rs7028891=G) and ((rs4979462=T) or (rs10739402=T))[a]	*ATP6V1G1; AKNA; DFNB31*	116-133	-26.160	1.749	3.1×10^{-17}	1.597	9.8×10^{-7}	1.696	2.7×10^{-22}	870 (36.0%)	1,413 (49.3%)	603 (61.7%)
9	(rs7028891=A) and ((rs2418376=A) or (rs10759773=G))[a]	*DEC1; TNC; LOC101928748*	153-189	-22.728	0.569	2.5×10^{-17}	0.674	2.3×10^{-5}	0.604	9.3×10^{-21}	873 (59.1%)	1,398 (46.7%)	615 (34.5%)
9	(rs10817678=G) or ((rs1407306=T) and (rs7048742=A))[a]	*ORM2*	111	-21.730	0.586	6.6×10^{-16}	0.672	3.2×10^{-5}	0.613	2.1×10^{-19}	960 (57.7%)	1,380 (46.7%)	546 (33.5%)
9	(rs4979462=T) or ((rs10739402=T) and (rs10817564=C))[a]	*COL27A1*	128	-12.015	1.687	4.7×10^{-15}	1.699	3.8×10^{-8}	1.687	1.5×10^{-21}	532 (33.1%)	1,390 (46.0%)	964 (58.6%)

[a] Contains no HLA region SNPs
Abbreviations: #, number

Supplementary Table S2: Component single SNPs and SNP pair haplotype effects for replicated 3-SNP haplotypes

Chr	3-SNP haplotype tree	Haplotype OR[a]	Haplotype P[a]	Single SNP	Alt. allele	SNP OR[b]	SNP P[b]	Component haplotype pair	Pair OR[c]	Pair P[c]
6	(rs3117106=C) and ((rs206018=G) or (rs9501179=A))	0.196	3.0×10^{-23}	rs3117106	C	0.433	1.2×10^{-20}	rs3117106=C and rs206018=G	0.154	0.002
				rs206018	G	0.924	0.276	rs3117106=C and rs9501179=A	0.203	5.2×10^{-21}
				rs9501179	A	0.903	0.117			
6	(rs3129881=C) or ((rs375244=A) and (rs3132947=G))	3.665	2.3×10^{-24}	rs3129881	C	2.227	1.8×10^{-23}	rs375244=A and rs3132947=G	1.238	7.9×10^{-5}
				rs375244	A	0.980	0.715			
				rs3132947	G	2.117	1.4×10^{-16}			
6	((rs35372932=T) and (rs9269190=C)) or (rs9270493=C)	0.365	9.6×10^{-32}	rs35372932	T	1.054	0.399	rs35372932=T and rs9269190=C	0.313	8.7×10^{-21}
				rs9269190	C	0.798	1.3×10^{-4}			
				rs9270493	C	0.488	8.0×10^{-11}			
6	(rs9268977=T) or ((rs3135395=T) or (rs550513=T))	3.448	1.2×10^{-29}	rs9268977	T	1.493	2.1×10^{-11}	rs9268977=T or rs3135395=T	3.136	3.8×10^{-29}
				rs3135395	T	1.149	0.010	rs9268977=T or rs550513=T	1.494	4.2×10^{-11}
				rs550513	T	0.858	0.112	rs3135395=T or rs550513=T	1.132	0.019
6	((rs9268634=G) or (rs35344500=C)) and (rs9275175=G)	0.448	1.3×10^{-35}	rs9268634	G	0.750	7.5×10^{-8}	rs9268634=G and rs9275175=G	0.467	7.6×10^{-31}
				rs35344500	C	0.497	1.2×10^{-9}	rs35344500=C and rs9275175=G	0.450	4.5×10^{-11}
				rs9275175	G	0.579	8.3×10^{-24}			
6	((rs9268831=T) or (rs9269190=T)) or (rs9270652=C)	3.075	7.3×10^{-29}	rs9268831	T	1.324	1.6×10^{-7}	rs9268831=T or rs9269190=T	1.633	2.8×10^{-16}
				rs9269190	T	1.252	1.3×10^{-4}	rs9268831=T or rs9270652=C	1.321	2.0×10^{-5}
				rs9270652	C	1.109	0.057	rs9269190=T or rs9270652=C	1.925	1.2×10^{-18}
6	(rs3132947=G) or ((rs9501179=G) and (rs41546114=C))	5.030	2.4×10^{-22}	rs3132947	G	2.117	1.4×10^{-16}	rs9501179=G and rs41546114=C	1.110	0.086
				rs9501179	G	1.107	0.117			
				rs41546114	C	0.957	0.685			
6	(rs9268831=T) or ((rs2395194=G) or (rs387608=A))	3.601	2.2×10^{-30}	rs9268831	T	1.324	1.6×10^{-7}	rs9268831=T or rs2395194=G	3.107	3.5×10^{-29}
				rs2395194	G	1.934	8.1×10^{-19}	rs9268831=T or rs387608=A	1.302	1.2×10^{-6}
				rs387608	A	1.095	0.170	rs2395194=G or rs387608=A	2.106	2.7×10^{-20}
6	(rs3132947=G) or ((rs9268014=C) and (rs41546114=C))	5.012	1.0×10^{-22}	rs3132947	G	2.117	1.4×10^{-16}	rs9268014=C and rs41546114=C	1.120	0.060
				rs9268014	C	1.119	0.078			
				rs41546114	C	0.957	0.685			
6	(rs3132947=G) or ((rs9268055=T) and (rs41546114=C))	5.012	1.0×10^{-22}	rs3132947	G	2.117	1.4×10^{-16}	rs9268055=T and rs41546114=C	1.134	0.036
				rs9268055	T	1.134	0.049			
				rs41546114	C	0.957	0.685			
6	((rs241437=G) or (rs3129299=C)) or (rs9276909=C)	4.716	1.8×10^{-20}	rs241437	G	1.441	6.1×10^{-12}	rs241437=G or rs3129299=C	1.903	6.7×10^{-15}
				rs3129299	C	1.225	4.9×10^{-4}	rs241437=G or rs9276909=C	3.413	4.2×10^{-21}
				rs9276909	C	1.438	1.7×10^{-8}	rs3129299=C or rs9276909=C	2.798	1.7×10^{-18}
6	((rs2395194=G) and (rs16870908=G)) or (rs9268831=T)	2.732	5.9×10^{-33}	rs2395194	G	1.934	8.1×10^{-19}	rs2395194=G and rs16870908=G	1.948	5.0×10^{-23}
				rs16870908	G	1.514	0.001			
				rs9268831	T	1.324	1.6×10^{-7}			
6	((rs9268213=A) and (rs41546114=C)) or (rs3132947=G)	5.012	1.0×10^{-22}	rs9268213	A	1.129	0.058	rs9268213=A and rs41546114=C	1.130	0.042
				rs41546114	C	0.957	0.685			
				rs3132947	G	2.117	1.4×10^{-16}			
6	((rs4538748=C) or (rs2064476=G)) and (rs35344500=A)	2.313	3.3×10^{-35}	rs4538748	C	1.519	5.1×10^{-13}	rs4538748=C and rs35344500=A	1.723	1.1×10^{-22}
				rs2064476	G	1.521	1.8×10^{-14}	rs2064476=G and rs35344500=A	1.623	2.0×10^{-19}
				rs35344500	A	2.013	1.2×10^{-9}			
6	((rs2244027=A) or (rs2242665=C)) or (rs2071591=G)	2.873	8.7×10^{-26}	rs2244027	A	1.316	4.7×10^{-7}	rs2244027=A or rs2242665=C	1.500	7.6×10^{-11}
				rs2242665	C	1.157	0.008	rs2244027=A or rs2071591=G	1.601	2.3×10^{-12}
				rs2071591	G	1.311	1.1×10^{-6}	rs2242665=C or rs2071591=G	1.787	5.2×10^{-16}
6	((rs9296088=A) and (rs12207818=C)) and (rs181997=A)	0.204	6.6×10^{-18}	rs9296088	A	0.752	6.1×10^{-8}	rs9296088=A and rs12207818=C	0.482	1.6×10^{-14}
				rs12207818	C	0.708	1.7×10^{-6}	rs9296088=A and rs181997=A	0.437	9.7×10^{-16}
				rs181997	A	0.619	3.3×10^{-10}	rs12207818=C and rs181997=A	0.284	1.3×10^{-16}
6	((rs2071287=C) and (rs3094596=C)) or (rs185819=C)	3.370	1.5×10^{-24}	rs2071287	C	1.482	7.1×10^{-12}	rs2071287=C and rs3094596=C	1.497	9.4×10^{-13}
				rs3094596	C	2.104	2.5×10^{-16}			
				rs185819	C	1.302	4.1×10^{-6}			

Chr	3-SNP haplotype tree	Haplotype OR[a]	Haplotype P[a]	Single SNP	Alt. allele	SNP OR[b]	SNP P[b]	Component haplotype pair	Pair OR[c]	Pair P[c]
6	(rs3132947=T) and ((rs404860=C) or (rs41546114=T))	0.253	3.2×10^{-21}	rs3132947	T	0.472	1.4×10^{-16}	rs3132947=T and rs404860=C	0.261	4.0×10^{-20}
				rs404860	C	1.115	0.036	rs3132947=T and rs41546114=T	0.333	0.055
				rs41546114	T	1.045	0.685			
6	(rs11754586=T) or ((rs3131932=G) or (rs9262537=G))	3.241	1.8×10^{-19}	rs11754586	T	1.090	0.125	rs11754586=T or rs3131932=G	1.205	0.002
				rs3131932	G	1.181	0.003	rs11754586=T or rs9262537=G	1.232	1.1×10^{-4}
				rs9262537	G	1.181	0.005	rs3131932=G or rs9262537=G	1.885	4.2×10^{-13}
6	((rs10947251=A) or (rs41546114=T)) and (rs3132947=T)	0.203	2.8×10^{-22}	rs10947251	A	0.891	0.075	rs10947251=A and rs3132947=T	0.199	1.5×10^{-21}
				rs41546114	T	1.045	0.685	rs41546114=T and rs3132947=T	0.333	0.055
				rs3132947	T	0.472	1.4×10^{-16}			
6	((rs3132947=G) or (rs9266632=G)) or (rs185819=C)	4.321	2.0×10^{-23}	rs3132947	G	2.117	1.4×10^{-16}	rs3132947=G or rs9266632=G	2.253	1.9×10^{-17}
				rs9266632	G	1.229	0.002	rs3132947=G or rs185819=C	3.733	1.2×10^{-22}
				rs185819	C	1.302	4.1×10^{-6}	rs9266632=G or rs185819=C	1.338	8.1×10^{-7}
6	(rs805273=A) and ((rs805267=G) or (rs9266774=T))	0.264	3.1×10^{-22}	rs805273	A	0.554	1.8×10^{-12}	rs805273=A and rs805267=G	0.275	2.4×10^{-19}
				rs805267	G	1.097	0.372	rs805273=A and rs9266774=T	0.214	2.1×10^{-4}
				rs9266774	T	0.984	0.774			
6	(rs3828901=A) or ((rs707921=A) and (rs9266774=T))	0.279	2.5×10^{-21}	rs3828901	A	0.292	2.0×10^{-18}	rs707921=A and rs9266774=T	0.201	3.2×10^{-4}
				rs707921	A	0.912	0.372			
				rs9266774	T	0.984	0.774			
6	(rs3828901=G) and ((rs805268=A) or (rs9266774=C))	3.586	4.4×10^{-21}	rs3828901	G	3.429	2.0×10^{-18}	rs3828901=G and rs805268=A	1.858	1.8×10^{-12}
				rs805268	A	1.085	0.466	rs3828901=G and rs9266774=C	1.293	3.6×10^{-6}
				rs9266774	C	1.016	0.774			
6	(rs3828901=A) or ((rs3828919=A) and (rs2523497=T))	0.313	9.0×10^{-20}	rs3828901	A	0.292	2.0×10^{-18}	rs3828919=A and rs2523497=T	0.535	0.029
				rs3828919	A	1.028	0.667			
				rs2523497	T	1.064	0.234			
6	(rs707922=G) or ((rs1046089=A) and (rs9266774=C))	3.748	6.2×10^{-22}	rs707922	G	1.794	2.9×10^{-12}	rs1046089=A and rs9266774=C	1.173	0.007
				rs1046089	A	1.059	0.306			
				rs9266774	C	1.016	0.774			
6	((rs3131932=G) or (rs28360042=C)) or (rs885950=C)	3.356	1.1×10^{-20}	rs3131932	G	1.181	0.003	rs3131932=G or rs28360042=C	2.826	2.2×10^{-19}
				rs28360042	C	1.165	0.005	rs3131932=G or rs885950=C	1.635	6.0×10^{-11}
				rs885950	C	1.165	0.004	rs28360042=C or rs885950=C	1.255	0.001
6	((rs9266774=T) and (rs2255741=A)) or (rs3828901=A)	0.279	2.6×10^{-21}	rs9266774	T	0.984	0.774	rs9266774=T and rs2255741=A	0.207	4.4×10^{-4}
				rs2255741	A	0.904	0.370			
				rs3828901	A	0.292	2.0×10^{-18}			
6	(rs3749946=A) and ((rs2517506=T) and (rs7741091=A))	0.287	1.9×10^{-20}	rs3749946	A	0.774	4.9×10^{-5}	rs3749946=A and rs2517506=T	0.363	7.8×10^{-17}
				rs2517506	T	0.921	0.157	rs3749946=A and rs7741091=A	0.709	6.1×10^{-7}
				rs7741091	A	0.852	0.004	rs2517506=T and rs7741091=A	0.831	4.9×10^{-4}
6	(rs3828901=G) and ((rs12110785=T) or (rs2523644=T))	3.544	6.3×10^{-20}	rs3828901	G	3.429	2.0×10^{-18}	rs3828901=G and rs12110785=T	1.322	8.1×10^{-6}
				rs12110785	T	1.315	1.3×10^{-5}	rs3828901=G and rs2523644=T	1.876	7.0×10^{-15}
				rs2523644	T	1.243	0.022			
6	((rs2239888=T) or (rs3134769=C)) and (rs3828901=G)	3.510	5.5×10^{-21}	rs2239888	T	0.894	0.119	rs2239888=T and rs3828901=G	1.278	1.8×10^{-4}
				rs3134769	C	1.091	0.272	rs3134769=C and rs3828901=G	1.578	1.4×10^{-10}
				rs3828901	G	3.429	2.0×10^{-18}			
6	(rs2517681=T) or ((rs4148248=C) and (rs2735078=A))	3.196	2.6×10^{-18}	rs2517681	T	0.956	0.386	rs4148248=C and rs2735078=A	1.274	4.4×10^{-6}
				rs4148248	C	2.975	1.1×10^{-16}			
				rs2735078	A	1.030	0.578			
6	(rs17195733=G) or ((rs13201129=C) and (rs1737069=T))	0.360	1.8×10^{-20}	rs17195733	G	0.333	6.0×10^{-17}	rs13201129=C and rs1737069=T	0.480	1.2×10^{-4}
				rs13201129	C	0.879	0.049			
				rs1737069	T	0.728	5.1×10^{-7}			
6	(rs3130785=T) and ((rs9261301=G) or (rs1264570=C))	0.340	2.7×10^{-17}	rs3130785	T	0.454	8.8×10^{-17}	rs3130785=T and rs9261301=G	0.421	0.053
				rs9261301	G	1.286	7.0×10^{-6}	rs3130785=T and rs1264570=C	0.333	3.1×10^{-17}
				rs1264570	C	0.813	1.3×10^{-4}			
6	(rs2517681=C) and ((rs3130785=T) or (rs2735078=G))	0.317	1.2×10^{-18}	rs2517681	C	1.046	0.386	rs2517681=C and rs3130785=T	0.330	2.9×10^{-17}
				rs3130785	T	0.454	8.8×10^{-17}	rs2517681=C and rs2735078=G	0.154	0.014
				rs2735078	G	0.971	0.578			

Chr	3-SNP haplotype tree	Haplotype OR[a]	Haplotype P[a]	Single SNP	Alt. allele	SNP OR[b]	SNP P[b]	Component haplotype pair	Pair OR[c]	Pair P[c]
6	((rs1345229=A) or (rs1233387=T)) or (rs1003581=G)	3.070	5.4×10^{-18}	rs1345229	A	1.341	0.001	rs1345229=A or rs1233387=T	1.159	0.006
				rs1233387	T	1.102	0.066	rs1345229=A or rs1003581=G	1.293	5.1×10^{-6}
				rs1003581	G	1.226	1.8×10^{-4}	rs1233387=T or rs1003581=G	2.653	4.6×10^{-16}
6	((rs4713429=G) or (rs12110785=T)) or (rs3131932=G)	3.093	1.0×10^{-19}	rs4713429	G	1.092	0.237	rs4713429=G or rs12110785=T	1.388	2.0×10^{-6}
				rs12110785	T	1.315	1.3×10^{-5}	rs4713429=G or rs3131932=G	1.337	1.4×10^{-5}
				rs3131932	G	1.181	0.003	rs12110785=T or rs3131932=G	1.911	1.4×10^{-13}
6	((rs1003581=G) and (rs16894681=T)) or (rs1233387=T)	2.541	5.5×10^{-17}	rs1003581	G	1.226	1.8×10^{-4}	rs1003581=G and rs16894681=T	1.253	2.3×10^{-5}
				rs16894681	T	1.009	0.928			
				rs1233387	T	1.102	0.066			
6	((rs2281043=T) or (rs7751451=G)) and (rs1635=A)	0.371	2.1×10^{-16}	rs2281043	T	0.746	8.7×10^{-5}	rs2281043=T and rs1635=A	0.358	7.5×10^{-15}
				rs7751451	G	0.974	0.789	rs7751451=G and rs1635=A	0.472	0.008
				rs1635	A	0.801	4.5×10^{-5}			
6	((rs3117192=C) or (rs16894216=T)) and (rs7773193=T)	2.711	2.0×10^{-16}	rs3117192	C	0.849	0.006	rs3117192=C and rs7773193=T	1.080	0.177
				rs16894216	T	0.856	0.077	rs16894216=T and rs7773193=T	1.310	2.2×10^{-4}
				rs7773193	T	2.697	1.5×10^{-14}			
6	(rs9295704=C) and ((rs2451752=A) and (rs2575174=C))	0.365	8.9×10^{-15}	rs9295704	C	0.669	1.5×10^{-7}	rs9295704=C and rs2451752=A	0.399	3.2×10^{-14}
				rs2451752	A	0.951	0.457	rs9295704=C and rs2575174=C	0.638	1.2×10^{-7}
				rs2575174	C	0.940	0.393	rs2451752=A and rs2575174=C	0.933	0.228
6	(rs6939576=G) or ((rs2859365=G) or (rs6930033=A))	2.820	1.7×10^{-16}	rs6939576	G	1.091	0.137	rs6939576=G or rs2859365=G	2.265	5.4×10^{-14}
				rs2859365	G	1.165	0.004	rs6939576=G or rs6930033=A	1.759	3.2×10^{-10}
				rs6930033	A	1.143	0.015	rs2859365=G or rs6930033=A	1.435	1.0×10^{-6}
6	(rs7773193=C) or ((rs17280818=T) and (rs2394100=T))	0.382	6.3×10^{-16}	rs7773193	C	0.371	1.5×10^{-14}	rs17280818=T and rs2394100=T	0.481	0.013
				rs17280818	T	0.988	0.904			
				rs2394100	T	1.034	0.602			
7	((rs12671658=T) or (rs12702656=A)) and (rs11768586=G)	0.066	3.9×10^{-9}	rs12671658	T	1.023	0.682	rs12671658=T and rs11768586=G	0.104	1.4×10^{-6}
				rs12702656	A	1.039	0.585	rs12702656=A and rs11768586=G	0.000	0.953
				rs11768586	G	0.865	0.010			
9	(rs4979484=C) or ((rs13300483=T) and (rs7028891=G))	1.672	3.0×10^{-21}	rs4979484	C	1.365	0.005	rs13300483=T and rs7028891=G	1.637	1.2×10^{-19}
				rs13300483	T	1.584	1.4×10^{-17}			
				rs7028891	G	1.574	2.8×10^{-17}			
9	(rs7028891=G) and ((rs4979462=T) or (rs10739402=T))	1.696	2.7×10^{-22}	rs7028891	G	1.574	2.8×10^{-17}	rs7028891=G and rs4979462=T	1.634	1.5×10^{-19}
				rs4979462	T	1.599	2.6×10^{-18}	rs7028891=G and rs10739402=T	1.387	1.5×10^{-5}
				rs10739402	T	1.193	0.003			
9	(rs7028891=A) and ((rs2418376=A) or (rs10759773=G))	0.604	9.3×10^{-21}	rs7028891	A	0.635	2.8×10^{-17}	rs7028891=A and rs2418376=A	0.628	7.5×10^{-18}
				rs2418376	A	1.003	0.972	rs7028891=A and rs10759773=G	0.735	4.5×10^{-7}
				rs10759773	G	0.921	0.118			
9	(rs10817678=G) or ((rs1407306=T) and (rs7048742=A))	0.613	2.1×10^{-19}	rs10817678	G	0.652	8.3×10^{-14}	rs1407306=T and rs7048742=A	0.710	3.6×10^{-4}
				rs1407306	T	0.785	0.003			
				rs7048742	A	1.166	0.014			
9	(rs4979462=T) or ((rs10739402=T) and (rs10817564=C))	1.687	1.5×10^{-21}	rs4979462	T	1.599	2.6×10^{-18}	rs10739402=T and rs10817564=C	1.258	0.004
				rs10739402	T	1.193	0.003			
				rs10817564	C	1.034	0.526			

[a] 3-SNP haplotype OR and p-value in the combined sample (N=2,886).
[b] Single SNP ORs and p-values, assuming an additive genetic effect model for the specified alternative allele.
[c] 2-SNP haplotype ORs and p-values, assuming an additive genetic effect model for the specified haplotype pattern.
Abbreviations: Alt, alternative.

Supplementary Table S3: Pre-phasing missingness rates for replicated 3-SNP haplotypes

	Entire sample (N=2,886)	All controls (N=1,505)	All cases (N=1,381)
Median, proportion missing	0.0104	0.0113	0.0109
IQR, proportion missing	0.0069	0.0086	0.0080
Max, proportion missing	0.0596	0.0585	0.0608

Per-sample tree missingness was defined as having at least 1 SNP genotype missing among the 3 SNPs constituting the haplotype for a given sample.

Supplementary Table S4: Comparison of distribution frequencies for replicated 3-SNP haplotypes in unphased and phased control groups in the Japan-PBC GWAS data and the phased 1000 Genomes Japanese reference panel

		PBC UNPHASED (controls, N=1,505)		PBC PHASED (controls, N=1,505)									1000 GENOMES, JPT (N=104)								
Chr	3-SNP haplotype tree (tree SNP order: rs1, rs2, rs3)	# Homozygous carriers	# Potential carriers of at least 1 copy	rs1 AF	rs2 AF	rs3 AF	h0	% (h0/N)	h1	% (h1/N)	h2	% (h2/N)	rs1 AF	rs2 AF	rs3 AF	h0	% (h0/N)	h1	% (h1/N)	h2	% (h2/N)
6	(rs3117106=C) and ((rs206018=G) or (rs9501179=A))	11	277	C=0.072	G=0.152	A=0.199	1,274	84.7%	218	14.5%	13	0.9%	C=0.173	G=0.159	A=0.192	86	82.7%	17	16.3%	1	1.0%
6	(rs3129881=C) or ((rs375244=A) and (rs3132947=G))	1,112	1,494	C=0.903	A=0.633	G=0.928	14	0.9%	282	18.7%	1,209	80.3%	C=0.784	A=0.615	G=0.875	3	2.9%	21	20.2%	80	76.9%
6	((rs35372932=T) and (rs9269190=C)) or (rs9270493=C)	36	755	T=0.243	C=0.704	C=0.047	987	65.6%	461	30.6%	57	3.8%	T=0.231	C=0.697	C=0.072	75	72.1%	27	26.0%	2	1.9%
6	(rs9268977=T) or ((rs3135395=T) or (rs550513=T))	841	1,469	T=0.760	T=0.407	T=0.074	36	2.4%	338	22.5%	1,131	75.1%	T=0.635	T=0.389	T=0.072	2	1.9%	26	25.0%	76	73.1%
6	((rs9268634=G) or (rs35344500=C)) and (rs9275175=G)	143	966	G=0.510	C=0.043	G=0.373	678	45.0%	661	43.9%	166	11.0%	G=0.615	C=0.067	G=0.476	50	48.1%	44	42.3%	10	9.6%
6	((rs9268831=T) or (rs9269190=T)) or (rs9270652=C)	857	1,466	T=0.579	T=0.296	C=0.653	39	2.6%	367	24.4%	1,099	73.0%	T=0.500	T=0.303	C=0.630	NA	NA	17	16.3%	87	83.7%
6	(rs3132947=G) or ((rs9501179=G) and (rs41546114=C))	1,247	1,493	G=0.928	G=0.801	C=0.936	11	0.7%	212	14.1%	1,282	85.2%	G=0.875	G=0.808	C=0.938	1	1.0%	15	14.4%	88	84.6%
6	(rs9268831=T) or ((rs2395194=G) or (rs387608=A))	1,039	1,468	T=0.579	G=0.883	A=0.199	35	2.3%	336	22.3%	1,134	75.3%	T=0.500	G=0.760	A=0.183	2	1.9%	26	25.0%	76	73.1%
6	(rs3132947=G) or ((rs9268014=C) and (rs41546114=C))	1,240	1,493	G=0.928	C=0.790	C=0.936	12	0.8%	215	14.3%	1,278	84.9%	G=0.875	C=0.798	C=0.938	1	1.0%	15	14.4%	88	84.6%
6	(rs3132947=G) or ((rs9268055=T) and (rs41546114=C))	1,242	1,493	G=0.928	T=0.793	C=0.936	12	0.8%	215	14.3%	1,278	84.9%	G=0.875	T=0.798	C=0.938	1	1.0%	15	14.4%	88	84.6%
6	((rs241437=G) or (rs3129299=C)) or (rs9276909=C)	1,218	1,489	G=0.593	C=0.727	C=0.808	16	1.1%	193	12.8%	1,296	86.1%	G=0.500	C=0.692	C=0.798	1	1.0%	13	12.5%	90	86.5%
6	((rs2395194=G) and (rs16870908=G)) or (rs9268831=T)	897	1,455	G=0.883	G=0.958	T=0.579	64	4.3%	475	31.6%	966	64.2%	G=0.760	G=0.947	T=0.500	3	2.9%	39	37.5%	62	59.6%
6	((rs9268213=A) and (rs41546114=C)) or (rs3132947=G)	1,240	1,493	A=0.791	C=0.936	G=0.928	12	0.8%	215	14.3%	1,278	84.9%	A=0.798	C=0.938	G=0.875	1	1.0%	15	14.4%	88	84.6%
6	((rs4538748=C) or (rs2064476=G)) and (rs35344500=A)	670	1,412	C=0.736	G=0.674	A=0.957	137	9.1%	604	40.1%	764	50.8%	C=0.601	G=0.558	A=0.933	4	3.8%	48	46.2%	52	50.0%
6	((rs2244027=A) or (rs2242665=C)) or (rs2071591=G)	875	1,478	A=0.632	C=0.386	G=0.668	26	1.7%	373	24.8%	1,106	73.5%	A=0.587	C=0.351	G=0.615	3	2.9%	21	20.2%	80	76.9%
6	((rs9296088=A) and (rs12207818=C)) and (rs181997=A)	9	209	A=0.539	C=0.138	A=0.110	1,326	88.1%	169	11.2%	10	0.7%	A=0.630	C=0.202	A=0.159	88	84.6%	16	15.4%	NA	NA
6	((rs2071287=C) and (rs3094596=C)) or (rs185819=C)	1,001	1,477	C=0.730	C=0.928	C=0.700	28	1.9%	284	18.9%	1,193	79.3%	C=0.615	C=0.846	C=0.688	2	1.9%	16	15.4%	86	82.7%
6	(rs3132947=T) and ((rs404860=C) or (rs41546114=T))	14	295	T=0.072	C=0.542	T=0.064	1,265	84.1%	226	15.0%	14	0.9%	T=0.125	C=0.481	T=0.062	87	83.7%	16	15.4%	1	1.0%
6	(rs11754586=T) or ((rs3131932=G) or (rs9262537=G))	811	1,493	T=0.346	G=0.703	G=0.298	12	0.8%	247	16.4%	1,246	82.8%	T=0.322	G=0.702	G=0.221	1	1.0%	16	15.4%	87	83.7%
6	((rs10947251=A) or (rs41546114=T)) and (rs3132947=T)	12	255	A=0.197	T=0.064	T=0.072	1,281	85.1%	212	14.1%	12	0.8%	A=0.192	T=0.062	T=0.125	88	84.6%	15	14.4%	1	1.0%
6	((rs3132947=G) or (rs9266632=G)) or (rs185819=C)	1,210	1,490	G=0.928	G=0.214	C=0.700	14	0.9%	238	15.8%	1,253	83.3%	G=0.875	G=0.139	C=0.688	2	1.9%	15	14.4%	87	83.7%
6	(rs805273=A) and ((rs805267=G) or (rs9266774=T))	10	403	A=0.088	G=0.936	T=0.304	1,245	82.7%	246	16.3%	14	0.9%	A=0.173	G=0.904	T=0.284	89	85.6%	13	12.5%	2	1.9%

		PBC UNPHASED (controls, N=1,505)		PBC PHASED (controls, N=1,505)									1000 GENOMES, JPT (N=104)								
Chr	3-SNP haplotype tree (tree SNP order: rs1, rs2, rs3)	# Homozygous carriers	# Potential carriers of at least 1 copy	rs1 AF	rs2 AF	rs3 AF	h0	% (h0/N)	h1	% (h1/N)	h2	% (h2/N)	rs1 AF	rs2 AF	rs3 AF	h0	% (h0/N)	h1	% (h1/N)	h2	% (h2/N)
6	(rs3828901=A) or ((rs707921=A) and (rs9266774=T))	9	303	A=0.025	A=0.064	T=0.304	1,245	82.7%	247	16.4%	13	0.9%	A=0.082	A=0.096	T=0.284	88	84.6%	14	13.5%	2	1.9%
6	(rs3828901=G) and ((rs805268=A) or (rs9266774=C))	1,209	1,494	G=0.975	A=0.946	C=0.696	12	0.8%	245	16.3%	1,248	82.9%	G=0.918	A=0.933	C=0.716	1	1.0%	15	14.4%	88	84.6%
6	(rs3828901=A) or ((rs3828919=A) and (rs2523497=T))	10	508	A=0.025	A=0.205	T=0.483	1,237	82.2%	258	17.1%	10	0.7%	A=0.082	A=0.197	T=0.452	86	82.7%	17	16.3%	1	1.0%
6	(rs707922=G) or ((rs1046089=A) and (rs9266774=C))	1,128	1,493	G=0.912	A=0.352	C=0.696	14	0.9%	244	16.2%	1,247	82.9%	G=0.827	A=0.370	C=0.716	2	1.9%	13	12.5%	89	85.6%
6	((rs3131932=G) or (rs28360042=C)) or (rs885950=C)	1,067	1,491	G=0.703	C=0.620	C=0.395	14	0.9%	252	16.7%	1,239	82.3%	G=0.702	C=0.577	C=0.317	1	1.0%	17	16.3%	86	82.7%
6	((rs9266774=T) and (rs2255741=A)) or (rs3828901=A)	9	295	T=0.304	A=0.054	A=0.025	1,245	82.7%	248	16.5%	12	0.8%	T=0.284	A=0.067	A=0.082	88	84.6%	15	14.4%	1	1.0%
6	(rs3749946=A) and ((rs2517506=T) and (rs7741091=A))	10	520	A=0.198	T=0.707	A=0.638	1,249	83.0%	246	16.3%	10	0.7%	A=0.250	T=0.668	A=0.678	87	83.7%	16	15.4%	1	1.0%
6	(rs3828901=G) and ((rs12110785=T) or (rs2523644=T))	1,218	1,494	G=0.975	T=0.789	T=0.927	10	0.7%	235	15.6%	1,260	83.7%	G=0.918	T=0.736	T=0.909	1	1.0%	15	14.4%	88	84.6%
6	((rs2239888=T) or (rs3134769=C)) and (rs3828901=G)	1,200	1,494	T=0.834	C=0.887	G=0.975	11	0.7%	250	16.6%	1,244	82.7%	T=0.856	C=0.856	G=0.918	1	1.0%	17	16.3%	86	82.7%
6	(rs2517681=T) or ((rs4148248=C) and (rs2735078=A))	797	1,489	T=0.493	C=0.971	A=0.624	12	0.8%	233	15.5%	1,260	83.7%	T=0.505	C=0.913	A=0.606	1	1.0%	17	16.3%	86	82.7%
6	(rs17195733=G) or ((rs13201129=C) and (rs1737069=T))	13	396	G=0.029	C=0.202	T=0.192	1,181	78.5%	305	20.3%	19	1.3%	G=0.091	C=0.173	T=0.255	83	79.8%	20	19.2%	1	1.0%
6	(rs3130785=T) and ((rs9261301=G) or (rs1264570=C))	12	323	T=0.065	G=0.377	C=0.419	1,253	83.3%	240	15.9%	12	0.8%	T=0.149	G=0.279	C=0.438	85	81.7%	18	17.3%	1	1.0%
6	(rs2517681=C) and ((rs3130785=T) or (rs2735078=G))	12	706	C=0.507	T=0.065	G=0.376	1,251	83.1%	242	16.1%	12	0.8%	C=0.495	T=0.149	G=0.394	85	81.7%	18	17.3%	1	1.0%
6	((rs1345229=A) or (rs1233387=T)) or (rs1003581=G)	964	1,489	A=0.123	T=0.561	G=0.680	13	0.9%	237	15.7%	1,255	83.4%	A=0.115	T=0.519	G=0.639	1	1.0%	16	15.4%	87	83.7%
6	((rs4713429=G) or (rs12110785=T)) or (rs3131932=G)	1,058	1,487	G=0.150	T=0.789	G=0.703	17	1.1%	255	16.9%	1,233	81.9%	G=0.120	T=0.736	G=0.702	1	1.0%	19	18.3%	84	80.8%
6	((rs1003581=G) and (rs16894681=T)) or (rs1233387=T)	901	1,484	G=0.680	T=0.927	T=0.561	17	1.1%	281	18.7%	1,207	80.2%	G=0.639	T=0.918	T=0.519	1	1.0%	22	21.2%	81	77.9%
6	((rs2281043=T) or (rs7751451=G)) and (rs1635=A)	12	386	T=0.129	G=0.074	A=0.346	1,243	82.6%	246	16.3%	16	1.1%	T=0.168	G=0.087	A=0.365	91	87.5%	12	11.5%	1	1.0%
6	((rs3117192=C) or (rs16894216=T)) and (rs7773193=T)	1,177	1,488	C=0.719	T=0.895	T=0.969	15	1.0%	246	16.3%	1,244	82.7%	C=0.716	T=0.889	T=0.918	1	1.0%	18	17.3%	85	81.7%
6	(rs9295704=C) and ((rs2451752=A) and (rs2575174=C))	10	385	C=0.114	A=0.806	C=0.849	1,274	84.7%	221	14.7%	10	0.7%	C=0.173	A=0.798	C=0.837	84	80.8%	19	18.3%	1	1.0%
6	(rs6939576=G) or ((rs2859365=G) or (rs6930033=A))	1,141	1,488	G=0.735	G=0.544	A=0.640	17	1.1%	230	15.3%	1,258	83.6%	G=0.707	G=0.524	A=0.601	1	1.0%	17	16.3%	86	82.7%
6	(rs7773193=C) or ((rs17280818=T) and (rs2394100=T))	14	300	C=0.031	T=0.075	T=0.209	1,243	82.6%	244	16.2%	18	1.2%	C=0.082	T=0.087	T=0.212	88	84.6%	15	14.4%	1	1.0%
7	((rs12671658=T) or (rs12702656=A)) and (rs11768586=G)	0	554	T=0.337	A=0.175	G=0.333	1,426	94.8%	79	5.2%	NA	NA	T=0.303	A=0.173	G=0.370	104	100.0%	NA	NA	NA	NA
9	(rs4979484=C) or ((rs13300483=T) and (rs7028891=G))	242	1,008	C=0.071	T=0.543	G=0.568	501	33.3%	736	48.9%	268	17.8%	C=0.058	T=0.438	G=0.452	33	31.7%	52	50.0%	19	18.3%

		PBC UNPHASED (controls, N=1,505)		PBC PHASED (controls, N=1,505)									1000 GENOMES, JPT (N=104)								
Chr	3-SNP haplotype tree (tree SNP order: rs1, rs2, rs3)	# Homozygous carriers	# Potential carriers of at least 1 copy	rs1 AF	rs2 AF	rs3 AF	h0	% (h0/N)	h1	% (h1/N)	h2	% (h2/N)	rs1 AF	rs2 AF	rs3 AF	h0	% (h0/N)	h1	% (h1/N)	h2	% (h2/N)
9	(rs7028891=G) and ((rs4979462=T) or (rs10739402=T))	221	982	G=0.568	T=0.580	T=0.292	557	37.0%	717	47.6%	231	15.3%	G=0.452	T=0.495	T=0.308	38	36.5%	46	44.2%	20	19.2%
9	(rs7028891=A) and ((rs2418376=A) or (rs10759773=G))	373	1,179	A=0.432	A=0.863	G=0.458	357	23.7%	745	49.5%	403	26.8%	A=0.548	A=0.851	G=0.462	29	27.9%	48	46.2%	27	26.0%
9	(rs10817678=G) or ((rs1407306=T) and (rs7048742=A))	224	1,137	G=0.292	T=0.108	A=0.774	406	27.0%	736	48.9%	363	24.1%	G=0.385	T=0.101	A=0.755	34	32.7%	47	45.2%	23	22.1%
9	(rs4979462=T) or ((rs10739402=T) and (rs10817564=C))	334	1,205	T=0.580	T=0.292	C=0.565	356	23.7%	750	49.8%	399	26.5%	T=0.495	T=0.308	C=0.596	19	18.3%	52	50.0%	33	31.7%

Abbreviations: rs1 AF, allele frequency (AF) for 1st SNP listed in 3-SNP haplotype; rs2 AF, AF for 2nd SNP in haplotype; rs3 AF, AF for 3rd SNP in haplotype; h0, number (#) carriers of 0 haplotype tree copies; h1, # carriers of 1 haplotype tree copy; h2, # carriers with 2 haplotype tree copies.

Supplementary Table S5: Application of the benchmark method to SNPs in replicated 3-SNP haplotypes identified with the proposed method

Chr	3-SNP haplotype tree	OR[a]	P[a]	Benchmark method (applied to logic tree SNPs only)[b]		
				Global score statistic	Degrees of freedom	P
6	(rs3117106=C) and ((rs206018=G) or (rs9501179=A))	0.196	$3.0x10^{-23}$	127.389	6	$4.6x10^{-25}$
6	(rs3129881=C) or ((rs375244=A) and (rs3132947=G))	3.665	$2.3x10^{-24}$	160.689	6	$4.2x10^{-32}$
6	((rs35372932=T) and (rs9269190=C)) or (rs9270493=C)	0.365	$9.6x10^{-32}$	158.755	5	$1.8x10^{-32}$
6	(rs9268977=T) or ((rs3135395=T) or (rs550513=T))	3.448	$1.2x10^{-29}$	119.847	7	$8.2x10^{-23}$
6	((rs9268634=G) or (rs35344500=C)) and (rs9275175=G)	0.448	$1.3x10^{-35}$	163.123	7	$7.1x10^{-32}$
6	((rs9268831=T) or (rs9269190=T)) or (rs9270652=C)	3.075	$7.3x10^{-29}$	140.289	6	$8.7x10^{-28}$
6	(rs3132947=G) or ((rs9501179=G) and (rs41546114=C))	5.030	$2.4x10^{-22}$	113.509	6	$3.8x10^{-22}$
6	(rs9268831=T) or ((rs2395194=G) or (rs387608=A))	3.601	$2.2x10^{-30}$	124.925	7	$7.2x10^{-24}$
6	(rs3132947=G) or ((rs9268014=C) and (rs41546114=C))	5.012	$1.0x10^{-22}$	113.035	6	$4.7x10^{-22}$
6	(rs3132947=G) or ((rs9268055=T) and (rs41546114=C))	5.012	$1.0x10^{-22}$	112.163	6	$7.2x10^{-22}$
6	((rs241437=G) or (rs3129299=C)) or (rs9276909=C)	4.716	$1.8x10^{-20}$	127.570	7	$2.0x10^{-24}$
6	((rs2395194=G) and (rs16870908=G)) or (rs9268831=T)	2.732	$5.9x10^{-33}$	146.693	6	$3.9x10^{-29}$
6	((rs9268213=A) and (rs41546114=C)) or (rs3132947=G)	5.012	$1.0x10^{-22}$	111.456	6	$1.0x10^{-21}$
6	((rs4538748=C) or (rs2064476=G)) and (rs35344500=A)	2.313	$3.3x10^{-35}$	168.448	6	$9.6x10^{-34}$
6	((rs2244027=A) or (rs2242665=C)) or (rs2071591=G)	2.873	$8.7x10^{-26}$	76.256	7	$8.0x10^{-14}$
6	((rs9296088=A) and (rs12207818=C)) and (rs181997=A)	0.204	$6.6x10^{-18}$	97.965	7	$2.8x10^{-18}$
6	((rs2071287=C) and (rs3094596=C)) or (rs185819=C)	3.370	$1.5x10^{-24}$	119.583	7	$9.4x10^{-23}$
6	(rs3132947=T) and ((rs404860=C) or (rs41546114=T))	0.253	$3.2x10^{-21}$	104.903	6	$2.4x10^{-20}$
6	(rs11754586=T) or ((rs3131932=G) or (rs9262537=G))	3.241	$1.8x10^{-19}$	76.098	6	$2.3x10^{-14}$
6	((rs10947251=A) or (rs41546114=T)) and (rs3132947=T)	0.203	$2.8x10^{-22}$	112.040	6	$7.6x10^{-22}$
6	((rs3132947=G) or (rs9266632=G)) or (rs185819=C)	4.321	$2.0x10^{-23}$	100.854	7	$7.2x10^{-19}$
6	(rs805273=A) and ((rs805267=G) or (rs9266774=T))	0.264	$3.1x10^{-22}$	101.022	5	$3.2x10^{-20}$
6	(rs3828901=A) or ((rs707921=A) and (rs9266774=T))	0.279	$2.5x10^{-21}$	94.802	5	$6.6x10^{-19}$
6	(rs3828901=G) and ((rs805268=A) or (rs9266774=C))	3.586	$4.4x10^{-21}$	92.605	5	$1.9x10^{-18}$
6	(rs3828901=A) or ((rs3828919=A) and (rs2523497=T))	0.313	$9.0x10^{-20}$	92.502	5	$2.0x10^{-18}$
6	(rs707922=G) or ((rs1046089=A) and (rs9266774=C))	3.748	$6.2x10^{-22}$	95.094	7	$1.1x10^{-17}$
6	((rs3131932=G) or (rs28360042=C)) or (rs885950=C)	3.356	$1.1x10^{-20}$	85.291	7	$1.1x10^{-15}$
6	((rs9266774=T) and (rs2255741=A)) or (rs3828901=A)	0.279	$2.6x10^{-21}$	93.411	5	$1.3x10^{-18}$
6	(rs3749946=A) and ((rs2517506=T) and (rs7741091=A))	0.287	$1.9x10^{-20}$	76.474	7	$7.2x10^{-14}$
6	(rs3828901=G) and ((rs12110785=T) or (rs2523644=T))	3.544	$6.3x10^{-20}$	97.538	4	$3.3x10^{-20}$
6	((rs2239888=T) or (rs3134769=C)) and (rs3828901=G)	3.510	$5.5x10^{-21}$	95.089	5	$5.7x10^{-19}$
6	(rs2517681=T) or ((rs4148248=C) and (rs2735078=A))	3.196	$2.6x10^{-18}$	92.509	4	$3.9x10^{-19}$
6	(rs17195733=G) or ((rs13201129=C) and (rs1737069=T))	0.360	$1.8x10^{-20}$	92.753	5	$1.8x10^{-18}$
6	(rs3130785=T) and ((rs9261301=G) or (rs1264570=C))	0.340	$2.7x10^{-17}$	92.694	6	$8.3x10^{-18}$
6	(rs2517681=C) and ((rs3130785=T) or (rs2735078=G))	0.317	$1.2x10^{-18}$	98.277	5	$1.2x10^{-19}$
6	((rs1345229=A) or (rs1233387=T)) or (rs1003581=G)	3.070	$5.4x10^{-18}$	73.057	7	$3.6x10^{-13}$
6	((rs4713429=G) or (rs12110785=T)) or (rs3131932=G)	3.093	$1.0x10^{-19}$	75.618	7	$1.1x10^{-13}$
6	((rs1003581=G) and (rs16894681=T)) or (rs1233387=T)	2.541	$5.5x10^{-17}$	64.413	6	$5.7x10^{-12}$
6	((rs2281043=T) or (rs7751451=G)) and (rs1635=A)	0.371	$2.1x10^{-16}$	55.683	7	$1.1x10^{-9}$
6	((rs3117192=C) or (rs16894216=T)) and (rs7773193=T)	2.711	$2.0x10^{-16}$	71.075	5	$6.1x10^{-14}$
6	(rs9295704=C) and ((rs2451752=A) and (rs2575174=C))	0.365	$8.9x10^{-15}$	53.502	7	$3.0x10^{-9}$
6	(rs6939576=G) or ((rs2859365=G) or (rs6930033=A))	2.820	$1.7x10^{-16}$	78.111	7	$3.3x10^{-14}$
6	(rs7773193=C) or ((rs17280818=T) and (rs2394100=T))	0.382	$6.3x10^{-16}$	67.438	5	$3.5x10^{-13}$

				Benchmark method (applied to logic tree SNPs only)[b]		
Chr	3-SNP haplotype tree	OR[a]	P[a]	Global score statistic	Degrees of freedom	P
7	((rs12671658=T) or (rs12702656=A)) and (rs11768586=G)	0.066	3.9×10^{-9}	31.899	6	1.7×10^{-5}
9	(rs4979484=C) or ((rs13300483=T) and (rs7028891=G))	1.672	3.0×10^{-21}	90.263	6	2.7×10^{-17}
9	(rs7028891=G) and ((rs4979462=T) or (rs10739402=T))	1.696	2.7×10^{-22}	100.579	7	8.2×10^{-19}
9	(rs7028891=A) and ((rs2418376=A) or (rs10759773=G))	0.604	9.3×10^{-21}	85.233	7	1.2×10^{-15}
9	(rs10817678=G) or ((rs1407306=T) and (rs7048742=A))	0.613	2.1×10^{-19}	91.584	6	1.4×10^{-17}
9	(rs4979462=T) or ((rs10739402=T) and (rs10817564=C))	1.687	1.5×10^{-21}	91.479	7	6.1×10^{-17}

[a] 3-SNP haplotype OR and p-value in the combined sample (N=2,886), under the proposed logic regression method.
[b] Benchmark haplotype association testing method implemented in R 'haplo.stats', with 3 SNPs specified in the haplotype tree (minimum haplotype frequency=20).

Supplementary Table S6: GTEx Analysis v7 eQTL enrichment analysis results for blood- and liver-related cell/tissue types

Cell/Tissue Type	Significant eQTLs, PBC SNPs (N=5,207)	Significant eQTLs, Comparison (N=76,136)	OR	Fisher P
EBV-transformed lymphocytes	64	618	1.521	$2.7x10^{-3}$
Whole blood	179	2,415	1.087	0.289
Liver	51	672	1.111	0.446

Abbreviations: EBV, Epstein-Barr virus; sig, significant; eQTL, expression quantitative trait loci.
Enrichment defined as any result meeting the Bonferroni-corrected p-value threshold (.05/3=0.017)

Supplementary Table S7: H3K4me1 histone mark enrichment analysis results for all 29 blood- and liver-related epigenomes

EID	EID Grouping	Epigenome Name	H3K4me1 Peak Overlap, PBC SNPs (N=106)	H3K4me1 Peak Overlap, Comparison (N=16,036)	OR	Fisher P
E032	HSC & B-cell	Primary B cells (from PB)	35	1,680	4.212	$4.1x10^{-10}$
E034	Blood & T-cell	Primary T cells from primary blood (from PB)	34	1,665	4.076	$1.3x10^{-9}$
E124	ENCODE2012	Monocytes-CD14+ RO01746	34	1,732	3.899	$3.7x10^{-9}$
E118	ENCODE2012	HepG2 hepatocellular carcinoma	31	1,538	3.895	$1.2x10^{-8}$
E029	HSC & B-cell	Primary monocytes (from PB)	24	1,412	3.031	$1.5x10^{-5}$
E044	Blood & T-cell	Primary T regulatory cells (from PB)	25	1,546	2.892	$2.2x10^{-5}$
E043	Blood & T-cell	Primary T helper cells (from PB)	28	1,863	2.731	$2.6x10^{-5}$
E038	Blood & T-cell	Primary T helper naive cells (from PB)	26	1,694	2.751	$4.2x10^{-5}$
E123	ENCODE2012	K562 leukemia	22	1,320	2.919	$5.0x10^{-5}$
E031	HSC & B-cell	Primary B cells from cord blood	20	1,163	2.974	$7.6x10^{-5}$
E041	Blood & T-cell	Primary T helper cells PMA-I stimulated	28	2,016	2.496	$1.1x10^{-4}$
E048	Blood & T-cell	Primary T CD8+ memory cells (from PB)	25	1,704	2.596	$1.1x10^{-4}$
E039	Blood & T-cell	Primary T helper naive cells (from PB)	25	1,751	2.518	$2.3x10^{-4}$
E037	Blood & T-cell	Primary T helper memory cells (from PB)	25	1,783	2.467	$2.7x10^{-4}$
E115	ENCODE2012	Dnd41 T cell leukemia	19	1,198	2.705	$3.3x10^{-4}$
E047	Blood & T-cell	Primary T CD8+ naive cells (from PB)	24	1,707	2.456	$3.7x10^{-4}$
E116	ENCODE2012	GM12878 lymphoblastoid	21	1,441	2.502	$5.1x10^{-4}$
E042	Blood & T-cell	Primary T helper 17 cells PMA-I stimulated	23	1,654	2.409	$5.8x10^{-4}$
E046	HSC & B-cell	Primary natural killer cells (from PB)	21	1,466	2.455	$6.3x10^{-4}$
E040	Blood & T-cell	Primary T helper memory cells (from PB)	23	1,672	2.380	$6.5x10^{-4}$
E030	HSC & B-cell	Primary neutrophils (from PB)	18	1,233	2.456	$1.4x10^{-3}$
E062	Blood & T-cell	Primary mononuclear cells (from PB)	14	913	2.520	0.005
E050	HSC & B-cell	Primary HSCs G-CSF-mobilized female	24	2,077	1.967	0.006
E051	HSC & B-cell	Primary HSCs G-CSF-mobilized male	21	1,766	1.996	0.008
E045	Blood & T-cell	Primary T cells effector/memory enriched (PB)	17	1,399	1.998	0.014
E066	Other	Liver	21	1,895	1.844	0.016
E036	HSC & B-cell	Primary HSCs short term culture	20	1,751	1.897	0.018
E035	HSC & B-cell	Primary HSCs	13	1,056	1.983	0.029
E033	Blood & T-cell	Primary T cells from cord blood	9	971	1.439	0.302

Abbreviations: H3K4me1, histone H3 lysine 4 monomethylation; EID, epigenome identifier; PB, peripheral blood; HSC, hematopoietic stem cell.
Enrichment defined as any result meeting the Bonferroni-corrected p-value threshold (.05/29=$1.7x10^{-3}$ for histone modification marks).

Supplementary Table S8: H3K4me3 histone mark enrichment analysis results for all 29 blood- and liver-related epigenomes

EID	EID Grouping	Epigenome Name	H3K4me3 Peak Overlap, PBC SNPs (N=106)	H3K4me3 Peak Overlap, Comparison (N=16,036)	OR	Fisher P
E032	HSC & B-cell	Primary B cells (from PB)	13	306	7.182	1.7×10^{-7}
E031	HSC & B-cell	Primary B cells from cord blood	13	335	6.550	4.5×10^{-7}
E116	ENCODE2012	GM12878 lymphoblastoid	18	699	4.487	9.0×10^{-7}
E062	Blood & T-cell	Primary mononuclear cells (from PB)	12	335	5.982	2.9×10^{-6}
E044	Blood & T-cell	Primary T regulatory cells (from PB)	15	575	4.431	7.0×10^{-6}
E124	ENCODE2012	Monocytes-CD14+ RO01746	19	896	3.690	7.1×10^{-6}
E115	ENCODE2012	Dnd41 T cell leukemia	12	389	5.134	1.3×10^{-5}
E034	Blood & T-cell	Primary T cells from primary blood (from PB)	11	357	5.084	3.0×10^{-5}
E118	ENCODE2012	HepG2 hepatocellular carcinoma	12	440	4.524	4.1×10^{-5}
E042	Blood & T-cell	Primary T helper 17 cells PMA-I stimulated	14	669	3.495	1.5×10^{-4}
E050	HSC & B-cell	Primary HSCs G-CSF-mobilized female	13	610	3.534	2.2×10^{-4}
E029	HSC & B-cell	Primary monocytes (from PB)	7	194	5.772	3.6×10^{-4}
E123	ENCODE2012	K562 leukemia	11	486	3.704	4.2×10^{-4}
E051	HSC & B-cell	Primary HSCs G-CSF-mobilized male	14	769	3.021	5.9×10^{-4}
E041	Blood & T-cell	Primary T helper cells PMA-I stimulated	14	772	3.008	6.2×10^{-4}
E046	HSC & B-cell	Primary natural killer cells (from PB)	8	283	4.543	6.6×10^{-4}
E035	HSC & B-cell	Primary HSCs	9	358	4.063	7.0×10^{-4}
E047	Blood & T-cell	Primary T CD8+ naive cells (from PB)	10	437	3.718	7.1×10^{-4}
E066	Other	Liver	14	786	2.952	7.3×10^{-4}
E030	HSC & B-cell	Primary neutrophils (from PB)	10	469	3.457	0.001
E037	Blood & T-cell	Primary T helper memory cells (from PB)	11	589	3.036	0.002
E038	Blood & T-cell	Primary T helper naive cells (from PB)	11	618	2.888	0.003
E033	Blood & T-cell	Primary T cells from cord blood	8	368	3.475	0.003
E043	Blood & T-cell	Primary T helper cells (from PB)	10	554	2.911	0.004
E048	Blood & T-cell	Primary T CD8+ memory cells (from PB)	11	656	2.714	0.004
E036	HSC & B-cell	Primary HSCs short term culture	9	517	2.785	0.008
E045	Blood & T-cell	Primary T cells effector/memory enriched (PB)	10	634	2.530	0.010
E039	Blood & T-cell	Primary T helper naive cells (from PB)	9	615	2.326	0.022
E040	Blood & T-cell	Primary T helper memory cells (from PB)	10	770	2.065	0.037

Abbreviations: H3K4me3, histone H3 lysine 4 trimethylation; EID, epigenome identifier; PB, peripheral blood; HSC, hematopoietic stem cell.
Enrichment defined as any result meeting the Bonferroni-corrected p-value threshold (.05/29=1.7×10^{-3} for histone modification marks).

Supplementary Table S9: DNase enrichment analysis results for all 11 blood- and liver-related epigenomes

EID	EID Grouping	Epigenome Name	DNase Peak Overlap, PBC SNPs (N=106)	DNase Peak Overlap, Comparison (N=16,036)	OR	Fisher P
E033	Blood & T-cell	Primary T cells from cord blood	8	337	3.802	0.002
E034	Blood & T-cell	Primary T cells from primary blood (from PB)	9	437	3.311	0.003
E124	ENCODE2012	Monocytes-CD14+ RO01746	9	439	3.296	0.003
E032	HSC & B-cell	Primary B cells (from PB)	9	463	3.120	0.004
E118	ENCODE2012	HepG2 hepatocellular carcinoma	6	319	2.956	0.020
E050	HSC & B-cell	Primary HSCs G-CSF-mobilized female	7	428	2.578	0.025
E116	ENCODE2012	GM12878 lymphoblastoid	8	581	2.171	0.059
E123	ENCODE2012	K562 leukemia	7	572	1.911	0.107
E046	HSC & B-cell	Primary natural killer cells (from PB)	5	397	1.950	0.194
E029	HSC & B-cell	Primary monocytes (from PB)	4	331	1.861	0.286
E051	HSC & B-cell	Primary HSCs G-CSF-mobilized male	4	352	1.747	0.300

Abbreviations: EID, epigenome identifier; PB, peripheral blood; HSC, hematopoietic stem cell.
Enrichment defined as any result meeting the Bonferroni-corrected p-value threshold (.05/11=4.5x10^{-3} for DNAse).

Supplementary Table S10: Functional annotations of SNPs in all replicated 3-SNP haplotypes

Chr	3-SNP Haplotype	SNP	Chr position (hg19)	Ontology	Mapped Gene	DHS overlap[a], # EIDs (PBC EIDs)	H3K4me1 overlap[a], # EIDs (PBC EIDs)	H3K4me3 overlap[a], # EIDs (PBC EIDs)	Bound protein[b]: Cell line (protein)	# Altered motifs[c]	Significant eQTLs[d]: Tissue (gene)
6	(rs3117106=C) and ((rs206018=G) or (rs9501179=A))	rs3117106	32343369	intergenic	*C6orf10*(dist=3680), *HCG23*(dist=14918)	0 (0)	11 (0)	3 (0)	NA	1	Whole Blood (*C4A, C4B, CYP21A1P, CYP21A2, HLA-DRB5, PBX2*); Lymphoblastoid (*C4A*); Liver (*C4A, CYP21A1P, HLA-DMA, STK19P, TNXA*)
6	(rs3117106=C) and ((rs206018=G) or (rs9501179=A))	rs206018	32177880	intronic	*NOTCH4*	0 (0)	8 (2)	2 (0)	NA	2	Whole Blood (*GPSM3|NOTCH4, HLA-DRA, LY6G5C, SKIV2L*)
6	(rs3117106=C) and ((rs206018=G) or (rs9501179=A))	rs9501179	32292993	intronic	*C6orf10*	0 (0)	2 (1)	2 (0)	NA	4	Whole Blood (*HLA-DRA*); Lymphoblastoid (*C2*)
6	(rs3129881=C) or ((rs375244=A) and (rs3132947=G))	rs3129881	32409484	intronic	*HLA-DRA*	0 (0)	42 (20)	50 (17)	GM12878 (OCT2, POL2, POL24H8, POU2F2); GM12891 (OCT2, POL2, POL24H8, POU2F2); GM12892 (POL2, POL24H8)	3	Whole Blood (*C4A, C4B, HLA-DQA1, HLA-DQA2, HLA-DQB2, HLA-DRB5*); Lymphoblastoid (*HLA-DQA2, HLA-DRB5*); Liver (*HLA-DRB5*)
6	(rs3129881=C) or ((rs375244=A) and (rs3132947=G))	rs375244	32191457	intronic	*NOTCH4*	0 (0)	69 (15)	38 (3)	NA	3	Whole Blood (*GPSM3|NOTCH4*)
6	(rs3129881=C) or ((rs375244=A) and (rs3132947=G))	rs3132947	32176782	intronic	*NOTCH4*	0 (0)	2 (1)	4 (0)	NA	3	Whole Blood (*AGPAT1, C4A, C4B, CYP21A1P, HLA-DRA, PBX2, SKIV2L*); Lymphoblastoid (*C4A, HLA-DQA1, RNF5*); Liver (*AGPAT1, RNF5*)
6	((rs35372932=T) and (rs9269190=C)) or (rs9270493=C)	rs35372932	32564985	intergenic	*HLA-DRB1*(dist=7372), *HLA-DQA1*(dist=40198)	0 (0)	1 (1)	0 (0)	NA	1	NA
6	((rs35372932=T) and (rs9269190=C)) or (rs9270493=C)	rs9269190	32448500	intergenic	*HLA-DRA*(dist=35674), *HLA-DRB5*(dist=36654)	2 (2)	4 (4)	2 (2)	NA	2	Whole Blood (*HLA-DRA*)
6	((rs35372932=T) and (rs9269190=C)) or (rs9270493=C)	rs9270493	32559110	intergenic	*HLA-DRB1*(dist=1497), *HLA-DQA1*(dist=46073)	0 (0)	12 (9)	2 (1)	NA	1	NA
6	(rs9268977=T) or ((rs3135395=T) or (rs550513=T))	rs9268977	32434939	intergenic	*HLA-DRA*(dist=22113), *HLA-DRB5*(dist=50215)	0 (0)	0 (0)	0 (0)	NA	0	Whole Blood (*C2, C4B, HLA-DRB5*); Liver (*C4A, HLA-DMA, HLA-DRB5*)
6	(rs9268977=T) or ((rs3135395=T) or (rs550513=T))	rs3135395	32405192	intergenic	*BTNL2*(dist=30285), *HLA-DRA*(dist=2427)	8 (8)	45 (19)	16 (8)	GM10847 (NFKB); GM12878 (NFKB); GM12891 (NFKB, POL2); GM12892 (NFKB, POL24H8); GM15510 (NFKB); GM18505 (NFKB); GM18951 (NFKB); GM19099 (NFKB); GM19193 (NFKB)	1	Whole Blood (*HLA-DQB1-AS1, HLA-DRB5, HLA-DRB6, HLA-DRB9*); Lymphoblastoid (*HLA-DRB5, HLA-DRB6, HLA-DRB9*); Liver (*HLA-DRB5*)
6	(rs9268977=T) or ((rs3135395=T) or (rs550513=T))	rs550513	31920687	intronic; downstream	*NELFE; CFB*	0 (0)	42 (7)	24 (2)	HepG2 (POL2)	0	Whole Blood (*HSPA1B, LY6G6F, RDBP, SKIV2L*); Lymphoblastoid (*SKIV2L*)

Chr	3-SNP Haplotype	SNP	Chr position (hg19)	Ontology	Mapped Gene	DHS overlap[a], # EIDs (PBC EIDs)	H3K4me1 overlap[a], # EIDs (PBC EIDs)	H3K4me3 overlap[a], # EIDs (PBC EIDs)	Bound protein[b]: Cell line (protein)	# Altered motifs[c]	Significant eQTLs[d]: Tissue (gene)
6	((rs9268634=G) or (rs35344500=C)) and (rs9275175=G)	rs9268634	32406530	intergenic	*BTNL2*(dist=31623), *HLA-DRA*(dist=1089)	1 (1)	30 (13)	14 (5)	NA	5	Whole Blood (*HLA-DQA1, HLA-DQA2, HLA-DQB1, HLA-DQB2, HLA-DRA, HLA-DRB1, HLA-DRB6*); Lymphoblastoid (*HLA-DQA2, HLA-DQB2, HLA-DRB6, HLA-DRB9, NOTCH4*); Liver (*HLA-DQA2*)
6	((rs9268634=G) or (rs35344500=C)) and (rs9275175=G)	rs35344500	32609525	intronic	*HLA-DQA1*	1 (1)	20 (15)	8 (6)	NA	0	Whole Blood (*HLA-DQA1, HLA-DQB1, HLA-DQB2, HLA-DRB1, HLA-DRB5, PSMB9*); Lymphoblastoid (*C4A, HLA-DQB2, HLA-DRB1, HLA-DRB9*); Liver (*C4A, HLA-DMA, HLA-DQB1, STK19P, TNXA*)
6	((rs9268634=G) or (rs35344500=C)) and (rs9275175=G)	rs9275175	32654147	intergenic	*HLA-DQB1*(dist=19681), *HLA-DQA2*(dist=55016)	0 (0)	26 (24)	7 (6)	NA	2	NA
6	((rs9268831=T) or (rs9269190=T)) or (rs9270652=C)	rs9268831	32427748	intergenic	*HLA-DRA*(dist=14922), *HLA-DRB5*(dist=57406)	5 (1)	31 (19)	71 (16)	GM18951 (POL2); MCF-7 (CMYC, HAE2F1, POL2)	1	Whole Blood (*HLA-DQA1, HLA-DQA2, HLA-DQB1, HLA-DQB1-AS1, HLA-DQB2, HLA-DRB1, HLA-DRB6, HLA-DRB9*); Lymphoblastoid (*HLA-DQA2, HLA-DQB2, HLA-DRB6, HLA-DRB9, NOTCH4*); Liver (*HLA-DQA2, HLA-DQB2*)
6	((rs9268831=T) or (rs9269190=T)) or (rs9270652=C)	rs9269190	32448500	intergenic	*HLA-DRA*(dist=35674), *HLA-DRB5*(dist=36654)	2 (2)	4 (4)	2 (2)	NA	2	Whole Blood (*HLA-DRA*)
6	((rs9268831=T) or (rs9269190=T)) or (rs9270652=C)	rs9270652	32565905	intergenic	*HLA-DRB1*(dist=8292), *HLA-DQA1*(dist=39278)	1 (1)	1 (1)	2 (0)	NA	0	NA
6	(rs3132947=G) or ((rs9501179=G) and (rs41546114=C))	rs3132947	32176782	intronic	*NOTCH4*	0 (0)	2 (1)	4 (0)	NA	3	Whole Blood (*AGPAT1, C4A, C4B, CYP21A1P, HLA-DRA, PBX2, SKIV2L*); Lymphoblastoid (*C4A, HLA-DQA1, RNF5*); Liver (*AGPAT1, RNF5*)
6	(rs3132947=G) or ((rs9501179=G) and (rs41546114=C))	rs9501179	32292993	intronic	*C6orf10*	0 (0)	2 (1)	2 (0)	NA	4	Whole Blood (*HLA-DRA*); Lymphoblastoid (*C2*)
6	(rs3132947=G) or ((rs9501179=G) and (rs41546114=C))	rs41546114	31382831	exonic; UTR3	*MICA*; *MICA*(NM_001289152:c.*72T>C, NM_001289153:c.*72T>C, NM_001289154:c.*72T>C, NM_001177519:c.*72T>C)	3 (1)	15 (8)	9 (3)	NA	0	NA
6	(rs9268831=T) or ((rs2395194=G) or (rs387608=A))	rs9268831	32427748	intergenic	*HLA-DRA*(dist=14922), *HLA-DRB5*(dist=57406)	5 (1)	31 (19)	71 (16)	GM18951 (POL2); MCF-7 (CMYC, HAE2F1, POL2)	1	Whole Blood (*HLA-DQA1, HLA-DQA2, HLA-DQB1, HLA-DQB1-AS1, HLA-DQB2, HLA-DRB1, HLA-DRB6, HLA-DRB9*); Lymphoblastoid (*HLA-DQA2, HLA-DQB2, HLA-DRB6, HLA-DRB9, NOTCH4*); Liver (*HLA-DQA2, HLA-DQB2*)
6	(rs9268831=T) or ((rs2395194=G) or (rs387608=A))	rs2395194	32447953	intergenic	*HLA-DRA*(dist=35127), *HLA-DRB5*(dist=37201)	0 (0)	0 (0)	0 (0)	NA	0	NA
6	(rs9268831=T) or ((rs2395194=G) or (rs387608=A))	rs387608	31941557	intronic	*STK19*	0 (0)	102 (23)	82 (14)	NA	4	Whole Blood (*HSPA1B, LSM2, RDBP, SKIV2L*); Lymphoblastoid (*SKIV2L*)
6	(rs3132947=G) or ((rs9268014=C) and (rs41546114=C))	rs3132947	32176782	intronic	*NOTCH4*	0 (0)	2 (1)	4 (0)	NA	3	Whole Blood (*AGPAT1, C4A, C4B, CYP21A1P, HLA-DRA, PBX2, SKIV2L*); Lymphoblastoid (*C4A, HLA-DQA1, RNF5*); Liver (*AGPAT1, RNF5*)

Chr	3-SNP Haplotype	SNP	Chr position (hg19)	Ontology	Mapped Gene	DHS overlap[a], # EIDs (PBC EIDs)	H3K4me1 overlap[a], # EIDs (PBC EIDs)	H3K4me3 overlap[a], # EIDs (PBC EIDs)	Bound protein[b]: Cell line (protein)	# Altered motifs[c]	Significant eQTLs[d]: Tissue (gene)
6	(rs3132947=G) or ((rs9268014=C) and (rs41546114=C))	rs9268014	32224852	intergenic	*NOTCH4*(dist=33008), *C6orf10*(dist=35623)	0 (0)	8 (2)	12 (0)	NA	0	Whole Blood (*C4B, HLA-DRB5*)
6	(rs3132947=G) or ((rs9268014=C) and (rs41546114=C))	rs41546114	31382831	exonic; UTR3	*MICA*; *MICA*(NM_001289152:c.*72T>C, NM_001289153:c.*72T>C, NM_001289154:c.*72T>C, NM_001177519:c.*72T>C)	3 (1)	15 (8)	9 (3)	NA	0	NA
6	(rs3132947=G) or ((rs9268055=T) and (rs41546114=C))	rs3132947	32176782	intronic	*NOTCH4*	0 (0)	2 (1)	4 (0)	NA	3	Whole Blood (*AGPAT1, C4A, C4B, CYP21A1P, HLA-DRA, PBX2, SKIV2L*); Lymphoblastoid (*C4A, HLA-DQA1, RNF5*); Liver (*AGPAT1, RNF5*)
6	(rs3132947=G) or ((rs9268055=T) and (rs41546114=C))	rs9268055	32230608	intergenic	*NOTCH4*(dist=38764), *C6orf10*(dist=29867)	0 (0)	0 (0)	0 (0)	NA	1	Whole Blood (*C4B, HLA-DRB5*); Liver (*C4A, CYP21A1P*)
6	(rs3132947=G) or ((rs9268055=T) and (rs41546114=C))	rs41546114	31382831	exonic; UTR3	*MICA*; *MICA*(NM_001289152:c.*72T>C, NM_001289153:c.*72T>C, NM_001289154:c.*72T>C, NM_001177519:c.*72T>C)	3 (1)	15 (8)	9 (3)	NA	0	NA
6	((rs241437=G) or (rs3129299=C)) or (rs9276909=C)	rs241437	32797684	intronic	*TAP2*	0 (0)	10 (7)	5 (1)	T-REx-HEK293 (ZNF263)	1	Whole Blood (*HLA-DMA, HLA-DOB, HLA-DRB5, PSMB9, TAP2*); Liver (*HLA-DRB5*)
6	((rs241437=G) or (rs3129299=C)) or (rs9276909=C)	rs3129299	32900787	intergenic	*LOC100294145*(dist=29252), *HLA-DMB*(dist=1619)	0 (0)	28 (11)	9 (2)	NA	2	Whole Blood (*HLA-DMA, TAP2*)
6	((rs241437=G) or (rs3129299=C)) or (rs9276909=C)	rs9276909	32850839	intergenic	*PSMB9*(dist=23211), *LOC100294145*(dist=11114)	13 (0)	29 (2)	9 (1)	MCF10A-Er-Src (STAT3)	0	Whole Blood (*HLA-DMA, HLA-DPB1, PSMB9, PSMB9*|*TAP1, TAP2*); Lymphoblastoid (*PSMB9*)
6	((rs2395194=G) and (rs16870908=G)) or (rs9268831=T)	rs2395194	32447953	intergenic	*HLA-DRA*(dist=35127), *HLA-DRB5*(dist=37201)	0 (0)	0 (0)	0 (0)	NA	0	NA
6	((rs2395194=G) and (rs16870908=G)) or (rs9268831=T)	rs16870908	32790089	exonic	*TAP2*	0 (0)	5 (3)	2 (2)	NA	1	Whole Blood (*HLA-DMB, HLA-DOA, HLA-DOB, PSMB8, PSMB9, TAP2*)
6	((rs2395194=G) and (rs16870908=G)) or (rs9268831=T)	rs9268831	32427748	intergenic	*HLA-DRA*(dist=14922), *HLA-DRB5*(dist=57406)	5 (1)	31 (19)	71 (16)	GM18951 (POL2); MCF-7 (CMYC, HAE2F1, POL2)	1	Whole Blood (*HLA-DQA1, HLA-DQA2, HLA-DQB1, HLA-DQB1-AS1, HLA-DQB2, HLA-DRB1, HLA-DRB6, HLA-DRB9*); Lymphoblastoid (*HLA-DQA2, HLA-DQB2, HLA-DRB6, HLA-DRB9, NOTCH4*); Liver (*HLA-DQA2, HLA-DQB2*)
6	((rs9268213=A) and (rs41546114=C)) or (rs3132947=G)	rs9268213	32282081	intronic	*C6orf10*	0 (0)	7 (0)	0 (0)	NA	2	Whole Blood (*C4A, C4B, HLA-DRB5*); Lymphoblastoid (*HLA-DQB1*); Liver (*C4A, CYP21A1P, HLA-DMA*)
6	((rs9268213=A) and (rs41546114=C)) or (rs3132947=G)	rs41546114	31382831	exonic; UTR3	*MICA*; *MICA*(NM_001289152:c.*72T>C, NM_001289153:c.*72T>C, NM_001289154:c.*72T>C, NM_001177519:c.*72T>C)	3 (1)	15 (8)	9 (3)	NA	0	NA

Chr	3-SNP Haplotype	SNP	Chr position (hg19)	Ontology	Mapped Gene	DHS overlap[a], # EIDs (PBC EIDs)	H3K4me1 overlap[a], # EIDs (PBC EIDs)	H3K4me3 overlap[a], # EIDs (PBC EIDs)	Bound protein[b]: Cell line (protein)	# Altered motifs[c]	Significant eQTLs[d]: Tissue (gene)
6	((rs9268213=A) and (rs41546114=C)) or (rs3132947=G)	rs3132947	32176782	intronic	*NOTCH4*	0 (0)	2 (1)	4 (0)	NA	3	Whole Blood (*AGPAT1, C4A, C4B, CYP21A1P, HLA-DRA, PBX2, SKIV2L*); Lymphoblastoid (*C4A, HLA-DQA1, RNF5*); Liver (*AGPAT1, RNF5*)
6	((rs4538748=C) or (rs2064476=G)) and (rs35344500=A)	rs4538748	32657505	intergenic	*HLA-DQB1*(dist=23039), *HLA-DQA2*(dist=51658)	5 (5)	26 (21)	6 (4)	GM10847 (NFKB); GM12878 (BATF, BCL11A, BCLAF1, IRF4, MEF2A, NFKB, OCT2, PAX5C20, PAX5N19, POL2, POL24H8, POU2F2, RFX5, SP1, SRF, TAF1, TBP, YY1); GM12891 (NFKB, OCT2, PAX5C20, POL2, POL24H8, POU2F2, TAF1, YY1); GM12892 (NFKB, PAX5C20, POL2, POL24H8, TAF1, YY1); GM15510 (NFKB); GM18505 (NFKB, POL2); GM18951 (NFKB); GM19099 (NFKB, POL2); Raji (POL2)	2	Whole Blood (*CYP21A1P, HLA-DOB, HLA-DQA1, HLA-DQA2, HLA-DQB1, HLA-DQB2*); Lymphoblastoid (*HLA-DQA2, HLA-DQB2, NOTCH4*); Liver (*HLA-DQB2*)
6	((rs4538748=C) or (rs2064476=G)) and (rs35344500=A)	rs2064476	33073322	intergenic	*HLA-DPB1*(dist=15849), *HLA-DPB2*(dist=6971)	0 (0)	8 (3)	3 (0)	NA	3	Whole Blood (*HLA-DPB2, RPL32P1*); Lymphoblastoid (*RPL32P1*)
6	((rs4538748=C) or (rs2064476=G)) and (rs35344500=A)	rs35344500	32609525	intronic	*HLA-DQA1*	1 (1)	20 (15)	8 (6)	NA	0	Whole Blood (*HLA-DQA1, HLA-DQB1, HLA-DQB2, HLA-DRB1, HLA-DRB5, PSMB9*); Lymphoblastoid (*C4A, HLA-DQB2, HLA-DRB1, HLA-DRB9*); Liver (*C4A, HLA-DMA, HLA-DQB1, STK19P, TNXA*)
6	((rs2244027=A) or (rs2242665=C)) or (rs2071591=G)	rs2244027	31347566	intergenic	*HLA-B*(dist=22577), *MICA*(dist=19995)	1 (0)	16 (0)	8 (1)	NA	2	Whole Blood (*HLA-C, MICA, MICB*)
6	((rs2244027=A) or (rs2242665=C)) or (rs2071591=G)	rs2242665	31839309	exonic	*SLC44A4*	0 (0)	55 (8)	22 (3)	NA	0	Whole Blood (*C6orf48, CSNK2B, CYP21A1P, HLA-DQA2, HLA-DQB2, HLA-DRB6, HSPA1B, RDBP, SKIV2L*); Lymphoblastoid (*SKIV2L*)
6	((rs2244027=A) or (rs2242665=C)) or (rs2071591=G)	rs2071591	31515799	intronic	*NFKBIL1*	1 (1)	120 (29)	123 (26)	NA	0	Whole Blood (*AIF1, BAT1, CSNK2B, DDX39B, HCP5, HLA-DRB5, LST1, TNF*)
6	((rs9296088=A) and (rs12207818=C)) and (rs181997=A)	rs9296088	33125742	intergenic	*HLA-DPB2*(dist=28852), *COL11A2*(dist=4727)	0 (0)	6 (2)	4 (2)	NA	6	NA
6	((rs9296088=A) and (rs12207818=C)) and (rs181997=A)	rs12207818	33809805	intergenic	*MLN*(dist=38012), *LINC01016*(dist=47483)	0 (0)	42 (10)	11 (1)	K562 (TFIIIC110)	1	NA
6	((rs9296088=A) and (rs12207818=C)) and (rs181997=A)	rs181997	32900718	intergenic	*LOC100294145*(dist=29183), *HLA-DMB*(dist=1688)	0 (0)	28 (11)	10 (2)	NA	4	Whole Blood (*HLA-DMA, TAP2*)
6	((rs2071287=C) and (rs3094596=C)) or (rs185819=C)	rs2071287	32170433	intronic	*NOTCH4*	1 (0)	17 (1)	8 (1)	NA	1	Whole Blood (*GPSM3|NOTCH4, HLA-DRA, SKIV2L*); Lymphoblastoid (*HLA-DQA1*); Liver (*AGPAT1*)
6	((rs2071287=C) and (rs3094596=C)) or (rs185819=C)	rs3094596	31350579	intergenic	*HLA-B*(dist=25590), *MICA*(dist=16982)	0 (0)	3 (0)	2 (0)	NA	3	Whole Blood (*AIF1, ATP6V1G2|BAT1, HCP5, LST1, LTA, MICB*)

Chr	3-SNP Haplotype	SNP	Chr position (hg19)	Ontology	Mapped Gene	DHS overlap[a], # EIDs (PBC EIDs)	H3K4me1 overlap[a], # EIDs (PBC EIDs)	H3K4me3 overlap[a], # EIDs (PBC EIDs)	Bound protein[b]: Cell line (protein)	# Altered motifs[c]	Significant eQTLs[d]: Tissue (gene)
6	((rs2071287=C) and (rs3094596=C)) or (rs185819=C)	rs185819	32050067	exonic	*TNXB*	2 (0)	70 (1)	34 (2)	NA	4	Whole Blood (*CYP21A1P, GPSM3|NOTCH4, HLA-DQA2, HLA-DQB2, HLA-DRB6, SKIV2L*); Liver (*CYP21A1P*)
6	(rs3132947=T) and ((rs404860=C) or (rs41546114=T))	rs3132947	32176782	intronic	*NOTCH4*	0 (0)	2 (1)	4 (0)	NA	3	Whole Blood (*AGPAT1, C4A, C4B, CYP21A1P, HLA-DRA, PBX2, SKIV2L*); Lymphoblastoid (*C4A, HLA-DQA1, RNF5*); Liver (*AGPAT1, RNF5*)
6	(rs3132947=T) and ((rs404860=C) or (rs41546114=T))	rs404860	32184345	intronic	*NOTCH4*	0 (0)	5 (1)	3 (0)	NA	0	NA
6	(rs3132947=T) and ((rs404860=C) or (rs41546114=T))	rs41546114	31382831	exonic; UTR3	*MICA*; *MICA*(NM_001289152:c.*72T>C, NM_001289153:c.*72T>C, NM_001289154:c.*72T>C, NM_001177519:c.*72T>C)	3 (1)	15 (8)	9 (3)	NA	0	NA
6	(rs11754586=T) or ((rs3131932=G) or (rs9262537=G))	rs11754586	31001911	intronic	*MUC22*	0 (0)	1 (0)	1 (0)	NA	3	Whole Blood (*VARSL*); Lymphoblastoid (*VARS2*)
6	(rs11754586=T) or ((rs3131932=G) or (rs9262537=G))	rs3131932	30940328	intergenic	*DPCR1*(dist=18330), *MUC21*(dist=11157)	0 (0)	9 (0)	5 (0)	NA	2	Whole Blood (*CCHCR1, FLOT1, IER3, LINC00243, VARSL*)
6	(rs11754586=T) or ((rs3131932=G) or (rs9262537=G))	rs9262537	30990224	intronic	*MUC22*	0 (0)	1 (0)	1 (0)	NA	1	Whole Blood (*HLA-S*)
6	((rs10947251=A) or (rs41546114=T)) and (rs3132947=T)	rs10947251	32261952	intronic	*C6orf10*	0 (0)	1 (0)	0 (0)	NA	1	Lymphoblastoid (*C2*)
6	((rs10947251=A) or (rs41546114=T)) and (rs3132947=T)	rs41546114	31382831	exonic; UTR3	*MICA*; *MICA*(NM_001289152:c.*72T>C, NM_001289153:c.*72T>C, NM_001289154:c.*72T>C, NM_001177519:c.*72T>C)	3 (1)	15 (8)	9 (3)	NA	0	NA
6	((rs10947251=A) or (rs41546114=T)) and (rs3132947=T)	rs3132947	32176782	intronic	*NOTCH4*	0 (0)	2 (1)	4 (0)	NA	3	Whole Blood (*AGPAT1, C4A, C4B, CYP21A1P, HLA-DRA, PBX2, SKIV2L*); Lymphoblastoid (*C4A, HLA-DQA1, RNF5*); Liver (*AGPAT1, RNF5*)
6	((rs3132947=G) or (rs9266632=G)) or (rs185819=C)	rs3132947	32176782	intronic	*NOTCH4*	0 (0)	2 (1)	4 (0)	NA	3	Whole Blood (*AGPAT1, C4A, C4B, CYP21A1P, HLA-DRA, PBX2, SKIV2L*); Lymphoblastoid (*C4A, HLA-DQA1, RNF5*); Liver (*AGPAT1, RNF5*)
6	((rs3132947=G) or (rs9266632=G)) or (rs185819=C)	rs9266632	31346902	intergenic	*HLA-B*(dist=21913), *MICA*(dist=20659)	1 (1)	17 (1)	11 (1)	NA	2	Whole Blood (*HLA-C, PSORS1C3*); Lymphoblastoid (*PSORS1C3*)
6	((rs3132947=G) or (rs9266632=G)) or (rs185819=C)	rs185819	32050067	exonic	*TNXB*	2 (0)	70 (1)	34 (2)	NA	4	Whole Blood (*CYP21A1P, GPSM3|NOTCH4, HLA-DQA2, HLA-DQB2, HLA-DRB6, SKIV2L*); Liver (*CYP21A1P*)
6	(rs805273=A) and ((rs805267=G) or (rs9266774=T))	rs805273	31665452	intronic	*ABHD16A*	1 (0)	52 (17)	24 (2)	NA	5	Whole Blood (*AIF1, LY6G5B, LY6G5C*)

Chr	3-SNP Haplotype	SNP	Chr position (hg19)	Ontology	Mapped Gene	DHS overlap[a], # EIDs (PBC EIDs)	H3K4me1 overlap[a], # EIDs (PBC EIDs)	H3K4me3 overlap[a], # EIDs (PBC EIDs)	Bound protein[b]: Cell line (protein)	# Altered motifs[c]	Significant eQTLs[d]: Tissue (gene)
6	(rs805273=A) and ((rs805267=G) or (rs9266774=T))	rs805267	31639757	exonic	*LY6G5B*	1 (0)	23 (8)	16 (2)	HepG2 (POL2)	3	Whole Blood (*AIF1, LY6G5B*)
6	(rs805273=A) and ((rs805267=G) or (rs9266774=T))	rs9266774	31352880	intergenic	*HLA-B*(dist=27891), *MICA*(dist=14681)	1 (1)	7 (0)	10 (1)	NA	5	Whole Blood (*HLA-C, LTA, MICA, MICB*)
6	(rs3828901=A) or ((rs707921=A) and (rs9266774=T))	rs3828901	31463718	intronic	*MICB*	8 (0)	101 (29)	94 (29)	HUVEC (CFOS)	1	NA
6	(rs3828901=A) or ((rs707921=A) and (rs9266774=T))	rs707921	31625541	intronic	*APOM*	4 (4)	112 (28)	70 (15)	HepG2 (POL2)	1	Whole Blood (*AIF1, LY6G5B*)
6	(rs3828901=A) or ((rs707921=A) and (rs9266774=T))	rs9266774	31352880	intergenic	*HLA-B*(dist=27891), *MICA*(dist=14681)	1 (1)	7 (0)	10 (1)	NA	5	Whole Blood (*HLA-C, LTA, MICA, MICB*)
6	(rs3828901=G) and ((rs805268=A) or (rs9266774=C))	rs3828901	31463718	intronic	*MICB*	8 (0)	101 (29)	94 (29)	HUVEC (CFOS)	1	NA
6	(rs3828901=G) and ((rs805268=A) or (rs9266774=C))	rs805268	31638178	upstream; downstream	*LY6G5B; CSNK2B*	0 (0)	42 (14)	37 (5)	NA	0	Whole Blood (*AIF1*)
6	(rs3828901=G) and ((rs805268=A) or (rs9266774=C))	rs9266774	31352880	intergenic	*HLA-B*(dist=27891), *MICA*(dist=14681)	1 (1)	7 (0)	10 (1)	NA	5	Whole Blood (*HLA-C, LTA, MICA, MICB*)
6	(rs3828901=A) or ((rs3828919=A) and (rs2523497=T))	rs3828901	31463718	intronic	*MICB*	8 (0)	101 (29)	94 (29)	HUVEC (CFOS)	1	NA
6	(rs3828901=A) or ((rs3828919=A) and (rs2523497=T))	rs3828919	31466057	intronic	*MICB*	12 (5)	111 (28)	125 (29)	GM12878 (POL2, ZEB1); GM12892 (POL2); GM19099 (POL2); H1-hESC (TAF1, TBP); HepG2 (HEY1, TAF1); K562 (CCNT2, ELF1, GABP, NRSF, POL2, ZBTB7A)	4	Whole Blood (*MICB*)
6	(rs3828901=A) or ((rs3828919=A) and (rs2523497=T))	rs2523497	31376928	intronic	*MICA*	0 (0)	60 (18)	5 (1)	NA	3	Whole Blood (*AIF1, HCG27, MICB, NOTCH4*); Lymphoblastoid (*MICA*); Liver (*MICA*)
6	(rs707922=G) or ((rs1046089=A) and (rs9266774=C))	rs707922	31625507	intronic	*APOM*	3 (3)	112 (28)	69 (15)	HepG2 (POL2)	5	Whole Blood (*AIF1, LY6G5B, LY6G5C*)
6	(rs707922=G) or ((rs1046089=A) and (rs9266774=C))	rs1046089	31602967	exonic	*PRRC2A*	0 (0)	38 (9)	23 (2)	HepG2 (POL2)	0	Whole Blood (*AIF1, C4A, C4B, HCP5, HLA-DRB5, HSPA1B, LY6G5B, LY6G5C*); Lymphoblastoid (*HLA-DRB5*); Liver (*HLA-DRB5*)
6	(rs707922=G) or ((rs1046089=A) and (rs9266774=C))	rs9266774	31352880	intergenic	*HLA-B*(dist=27891), *MICA*(dist=14681)	1 (1)	7 (0)	10 (1)	NA	5	Whole Blood (*HLA-C, LTA, MICA, MICB*)

Chr	3-SNP Haplotype	SNP	Chr position (hg19)	Ontology	Mapped Gene	DHS overlap[a], # EIDs (PBC EIDs)	H3K4me1 overlap[a], # EIDs (PBC EIDs)	H3K4me3 overlap[a], # EIDs (PBC EIDs)	Bound protein[b]: Cell line (protein)	# Altered motifs[c]	Significant eQTLs[d]: Tissue (gene)
6	((rs3131932=G) or (rs28360042=C)) or (rs885950=C)	rs3131932	30940328	intergenic	*DPCR1*(dist=18330), *MUC21*(dist=11157)	0 (0)	9 (0)	5 (0)	NA	2	Whole Blood (*CCHCR1, FLOT1, IER3, LINC00243, VARSL*)
6	((rs3131932=G) or (rs28360042=C)) or (rs885950=C)	rs28360042	31001781	intronic	*MUC22*	0 (0)	1 (0)	1 (0)	NA	4	Whole Blood (*HCG27, HLA-C, POU5F1, PSORS1C3*); Lymphoblastoid (*CCHCR1, HLA-C, PSORS1C3, TCF19*); Liver (*HLA-C, PSORS1C3*)
6	((rs3131932=G) or (rs28360042=C)) or (rs885950=C)	rs885950	31140152	intergenic	*POU5F1*(dist=1682), *PSORS1C3*(dist=1360)	5 (0)	40 (0)	25 (2)	H1-hESC (POL2)	6	Whole Blood (*HCG27, VARSL*)
6	((rs9266774=T) and (rs2255741=A)) or (rs3828901=A)	rs9266774	31352880	intergenic	*HLA-B*(dist=27891), *MICA*(dist=14681)	1 (1)	7 (0)	10 (1)	NA	5	Whole Blood (*HLA-C, LTA, MICA, MICB*)
6	((rs9266774=T) and (rs2255741=A)) or (rs3828901=A)	rs2255741	31605167	intronic	*PRRC2A*	12 (3)	45 (7)	14 (0)	GM12878 (POL2); K562 (POL2, POL24H8)	2	Whole Blood (*AIF1*)
6	((rs9266774=T) and (rs2255741=A)) or (rs3828901=A)	rs3828901	31463718	intronic	*MICB*	8 (0)	101 (29)	94 (29)	HUVEC (CFOS)	1	NA
6	(rs3749946=A) and ((rs2517506=T) and (rs7741091=A))	rs3749946	31448862	intergenic	*HCG26*(dist=8677), *MICB*(dist=13796)	0 (0)	8 (3)	3 (1)	NA	3	Whole Blood (*HCP5, LST1, MICB*)
6	(rs3749946=A) and ((rs2517506=T) and (rs7741091=A))	rs2517506	31031680	intergenic	*HCG22*(dist=4025), *C6orf15*(dist=47320)	0 (0)	13 (0)	1 (1)	NA	1	Whole Blood (*CCHCR1, FLOT1, HLA-L, MICB*)
6	(rs3749946=A) and ((rs2517506=T) and (rs7741091=A))	rs7741091	31352631	intergenic	*HLA-B*(dist=27642), *MICA*(dist=14930)	0 (0)	7 (0)	10 (1)	NA	2	Whole Blood (*ATP6V1G2|BAT1, HCG27, HLA-C, HLA-S, MICA, MICB, NOTCH4, ZBTB12*); Liver (*MICA*)
6	(rs3828901=G) and ((rs12110785=T) or (rs2523644=T))	rs3828901	31463718	intronic	*MICB*	8 (0)	101 (29)	94 (29)	HUVEC (CFOS)	1	NA
6	(rs3828901=G) and ((rs12110785=T) or (rs2523644=T))	rs12110785	30997824	exonic	*MUC22*	0 (0)	3 (0)	3 (0)	NA	1	NA
6	(rs3828901=G) and ((rs12110785=T) or (rs2523644=T))	rs2523644	31342484	intergenic	*HLA-B*(dist=17495), *MICA*(dist=25077)	0 (0)	2 (0)	2 (0)	HeLa-S3 (CTCF)	2	Whole Blood (*AIF1, ATP6V1G2|BAT1, HCG27, HCP5, LTA, MICB*); Lymphoblastoid (*HCG27*); Liver (*HLA-C*)
6	((rs2239888=T) or (rs3134769=C)) and (rs3828901=G)	rs2239888	30649912	intronic	*PPP1R18*	20 (6)	125 (29)	120 (29)	GM12878 (PU1); GM12891 (PU1); HUVEC (CFOS); HeLa-S3 (INI1, JUND, STAT1, TBP); HepG2 (JUND); K562 (CJUN, CMYC, FOSL1, JUNB, POL2, STAT1, STAT2, TAF1, ZBTB7A); MCF10A-Er-Src (STAT3)	2	NA
6	((rs2239888=T) or (rs3134769=C)) and (rs3828901=G)	rs3134769	31205754	intergenic	*HCG27*(dist=34009), *HLA-C*(dist=30772)	0 (0)	0 (0)	0 (0)	NA	1	Whole Blood (*CCHCR1, HCG27*); Lymphoblastoid (*C4B*); Liver (*HCG27*)

Chr	3-SNP Haplotype	SNP	Chr position (hg19)	Ontology	Mapped Gene	DHS overlap[a], # EIDs (PBC EIDs)	H3K4me1 overlap[a], # EIDs (PBC EIDs)	H3K4me3 overlap[a], # EIDs (PBC EIDs)	Bound protein[b]: Cell line (protein)	# Altered motifs[c]	Significant eQTLs[d]: Tissue (gene)
6	((rs2239888=T) or (rs3134769=C)) and (rs3828901=G)	rs3828901	31463718	intronic	*MICB*	8 (0)	101 (29)	94 (29)	HUVEC (CFOS)	1	NA
6	(rs2517681=T) or ((rs4148248=C) and (rs2735078=A))	rs2517681	29932330	intergenic	*HLA-A*(dist=18669), *HCG9*(dist=10562)	2 (0)	122 (29)	94 (25)	HTB-11 (NRSF)	1	Whole Blood (*HCG4P3, HCG4P5, HLA-A, HLA-F, HLA-G, HLA-J, HLA-V, IFITM4P, MICD, PPP1R11, ZFP57, ZNRD1*); Lymphoblastoid (*HCG4P5, IFITM4P, MICE*); Liver (*ZFP57*)
6	(rs2517681=T) or ((rs4148248=C) and (rs2735078=A))	rs4148248	30557566	intronic	*ABCF1*	0 (0)	39 (6)	12 (0)	NA	5	NA
6	(rs2517681=T) or ((rs4148248=C) and (rs2735078=A))	rs2735078	29941400	intergenic	*HLA-A*(dist=27739), *HCG9*(dist=1492)	0 (0)	23 (9)	10 (2)	NA	0	Whole Blood (*DDX39BP2, GABBR1, HCG4P3, HCG4P5, HLA-A, HLA-H, HLA-W, RANP1, ZFP57*); Lymphoblastoid (*HCG4P5, HCG4P7, HLA-A, HLA-H, HLA-K, ZFP57*); Liver (*HCG4B, HCG4P3, HLA-K, ZFP57*)
6	(rs17195733=G) or ((rs13201129=C) and (rs1737069=T))	rs17195733	30716991	intergenic	*IER3*(dist=4664), *LINC00243*(dist=63652)	17 (1)	96 (11)	41 (5)	A549 (GR, POL2); HCT-116 (POL24H8); HUVEC (CJUN, GATA2, POL2); HeLa-S3 (POL2); HepG2 (ELF1, FOSL2, FOXA1, FOXA2, HDAC2, HEY1, P300, POL2, RXRA, SP1)	5	NA
6	(rs17195733=G) or ((rs13201129=C) and (rs1737069=T))	rs13201129	30601067	intronic	*ATAT1*	0 (0)	8 (1)	6 (1)	NA	1	Whole Blood (*HLA-E, IFITM4P, MRPS18B, NRM*)
6	(rs17195733=G) or ((rs13201129=C) and (rs1737069=T))	rs1737069	29730730	intergenic	*IFITM4P*(dist=11805), *HCG4*(dist=28078)	3 (0)	50 (2)	10 (1)	GM15510 (NFKB)	3	Whole Blood (*AL645939.6-3, GABBR1, HCG9, HLA-F, HLA-F-AS1, HLA-G, HLA-H, HLA-J, IFITM4P*); Lymphoblastoid (*HLA-F, HLA-F-AS1, IFITM4P*); Liver (*HLA-A, HLA-F*)
6	(rs3130785=T) and ((rs9261301=G) or (rs1264570=C))	rs3130785	30796738	ncRNA_intronic	*LINC00243*	10 (2)	121 (29)	82 (25)	NA	5	Whole Blood (*DDR1, FLOT1, HCG9, HLA-H, HLA-J, HLA-L, IER3, VARS2, VARSL*); Lymphoblastoid (*HLA-J*); Liver (*HLA-H*)
6	(rs3130785=T) and ((rs9261301=G) or (rs1264570=C))	rs9261301	30041559	intronic	*RNF39*	0 (0)	67 (11)	47 (3)	NA	2	Whole Blood (*HCG4P3, HLA-A, HLA-G, HLA-L, HLA-V, PPP1R11, RPL23AP1, ZFP57*); Lymphoblastoid (*RPL23AP1*); Liver (*HLA-V, MICE*)
6	(rs3130785=T) and ((rs9261301=G) or (rs1264570=C))	rs1264570	30365210	intergenic	*TRIM39-RPP21*(dist=50575), *HLA-E*(dist=91973)	1 (1)	37 (12)	14 (1)	NA	2	Whole Blood (*HLA-E, MRPS18B, RPP21*); Liver (*HCG4B*)
6	(rs2517681=C) and ((rs3130785=T) or (rs2735078=G))	rs2517681	29932330	intergenic	*HLA-A*(dist=18669), *HCG9*(dist=10562)	2 (0)	122 (29)	94 (25)	HTB-11 (NRSF)	1	Whole Blood (*HCG4P3, HCG4P5, HLA-A, HLA-F, HLA-G, HLA-J, HLA-V, IFITM4P, MICD, PPP1R11, ZFP57, ZNRD1*); Lymphoblastoid (*HCG4P5, IFITM4P, MICE*); Liver (*ZFP57*)
6	(rs2517681=C) and ((rs3130785=T) or (rs2735078=G))	rs3130785	30796738	ncRNA_intronic	*LINC00243*	10 (2)	121 (29)	82 (25)	NA	5	Whole Blood (*DDR1, FLOT1, HCG9, HLA-H, HLA-J, HLA-L, IER3, VARS2, VARSL*); Lymphoblastoid (*HLA-J*); Liver (*HLA-H*)

Chr	3-SNP Haplotype	SNP	Chr position (hg19)	Ontology	Mapped Gene	DHS overlap[a], # EIDs (PBC EIDs)	H3K4me1 overlap[a], # EIDs (PBC EIDs)	H3K4me3 overlap[a], # EIDs (PBC EIDs)	Bound protein[b]: Cell line (protein)	# Altered motifs[c]	Significant eQTLs[d]: Tissue (gene)
6	(rs2517681=C) and ((rs3130785=T) or (rs2735078=G))	rs2735078	29941400	intergenic	*HLA-A*(dist=27739), *HCG9*(dist=1492)	0 (0)	23 (9)	10 (2)	NA	0	Whole Blood (*DDX39BP2, GABBR1, HCG4P3, HCG4P5, HLA-A, HLA-H, HLA-W, RANP1, ZFP57*); Lymphoblastoid (*HCG4P5, HCG4P7, HLA-A, HLA-H, HLA-K, ZFP57*); Liver (*HCG4B, HCG4P3, HLA-K, ZFP57*)
6	((rs1345229=A) or (rs1233387=T)) or (rs1003581=G)	rs1345229	30182395	intergenic	*TRIM26*(dist=1124), *HCG17*(dist=19421)	35 (10)	118 (27)	127 (29)	GM12878 (TBP)	4	Whole Blood (*TRIM10, ZNRD1*)
6	((rs1345229=A) or (rs1233387=T)) or (rs1003581=G)	rs1233387	29555864	exonic	*OR2H2*	0 (0)	1 (0)	3 (0)	NA	0	Whole Blood (*HLA-F, HLA-G, TRIM27*); Liver (*HLA-F*)
6	((rs1345229=A) or (rs1233387=T)) or (rs1003581=G)	rs1003581	29540204	intergenic	*UBD*(dist=12502), *SNORD32B*(dist=9825)	0 (0)	0 (0)	0 (0)	NA	3	Whole Blood (*HLA-F*)
6	((rs4713429=G) or (rs12110785=T)) or (rs3131932=G)	rs4713429	31021017	upstream	*HCG22*	2 (2)	18 (4)	11 (4)	GM12878 (EBF1)	7	Whole Blood (*VARSL*); Lymphoblastoid (*TCF19*)
6	((rs4713429=G) or (rs12110785=T)) or (rs3131932=G)	rs12110785	30997824	exonic	*MUC22*	0 (0)	3 (0)	3 (0)	NA	1	NA
6	((rs4713429=G) or (rs12110785=T)) or (rs3131932=G)	rs3131932	30940328	intergenic	*DPCR1*(dist=18330), *MUC21*(dist=11157)	0 (0)	9 (0)	5 (0)	NA	2	Whole Blood (*CCHCR1, FLOT1, IER3, LINC00243, VARSL*)
6	((rs1003581=G) and (rs16894681=T)) or (rs1233387=T)	rs1003581	29540204	intergenic	*UBD*(dist=12502), *SNORD32B*(dist=9825)	0 (0)	0 (0)	0 (0)	NA	3	Whole Blood (*HLA-F*)
6	((rs1003581=G) and (rs16894681=T)) or (rs1233387=T)	rs16894681	29232072	intergenic	*OR2J2*(dist=89721), *OR14J1*(dist=42395)	0 (0)	0 (0)	0 (0)	NA	4	NA
6	((rs1003581=G) and (rs16894681=T)) or (rs1233387=T)	rs1233387	29555864	exonic	*OR2H2*	0 (0)	1 (0)	3 (0)	NA	0	Whole Blood (*HLA-F, HLA-G, TRIM27*); Liver (*HLA-F*)
6	((rs2281043=T) or (rs7751451=G)) and (rs1635=A)	rs2281043	28268497	intronic	*PGBD1*	0 (0)	3 (1)	2 (1)	NA	3	Whole Blood (*TRIM27, ZKSCAN3, ZNF193*)
6	((rs2281043=T) or (rs7751451=G)) and (rs1635=A)	rs7751451	28752883	intergenic	*ZBED9*(dist=197771), *LINC01623*(dist=74519)	1 (1)	6 (0)	6 (0)	NA	4	Whole Blood (*ZFP57*)
6	((rs2281043=T) or (rs7751451=G)) and (rs1635=A)	rs1635	28227604	exonic; upstream	*NKAPL; ZKSCAN4*	0 (0)	45 (6)	72 (11)	NA	0	NA
6	((rs3117192=C) or (rs16894216=T)) and (rs7773193=T)	rs3117192	29401416	intergenic	*OR11A1*(dist=5907), *OR10C1*(dist=6300)	0 (0)	0 (0)	0 (0)	NA	5	NA

Chr	3-SNP Haplotype	SNP	Chr position (hg19)	Ontology	Mapped Gene	DHS overlap[a], # EIDs (PBC EIDs)	H3K4me1 overlap[a], # EIDs (PBC EIDs)	H3K4me3 overlap[a], # EIDs (PBC EIDs)	Bound protein[b]: Cell line (protein)	# Altered motifs[c]	Significant eQTLs[d]: Tissue (gene)
6	((rs3117192=C) or (rs16894216=T)) and (rs7773193=T)	rs16894216	28664213	intergenic	*ZBED9*(dist=109101), *LINC01623*(dist=163189)	4 (0)	33 (6)	47 (1)	NA	3	NA
6	((rs3117192=C) or (rs16894216=T)) and (rs7773193=T)	rs7773193	28611334	intergenic	*ZBED9*(dist=56222), *LINC01623*(dist=216068)	31 (4)	6 (0)	24 (0)	A549 (USF1); GM12878 (TBP); H1-hESC (TBP); HEK293(b) (KAP1); HeLa-S3 (AP2GAMMA, BRCA1, CEBPB, RFX5, RPC155, STAT1, TBP, TFIIIC110); HepG2 (CEBPB, HSF1, TBP); K562 (RPC155, TBP, TFIIIC110)	2	NA
6	(rs9295704=C) and ((rs2451752=A) and (rs2575174=C))	rs9295704	26704816	intergenic	*ZNF322*(dist=44836), *GUSBP2*(dist=134450)	0 (0)	13 (1)	8 (1)	NA	4	Whole Blood (*ABT1*)
6	(rs9295704=C) and ((rs2451752=A) and (rs2575174=C))	rs2451752	26648013	intronic	*ZNF322*	0 (0)	1 (1)	3 (1)	NA	0	Whole Blood (*BTN3A1, BTN3A2, HMGN4, ZNF322*)
6	(rs9295704=C) and ((rs2451752=A) and (rs2575174=C))	rs2575174	25885552	intergenic	*SLC17A3*(dist=11081), *SLC17A2*(dist=27432)	0 (0)	5 (0)	0 (0)	NA	2	Whole Blood (*HIST1H1T*|*HIST1H4A*)
6	(rs6939576=G) or ((rs2859365=G) or (rs6930033=A))	rs6939576	28669315	intergenic	*ZBED9*(dist=114203), *LINC01623*(dist=158087)	0 (0)	2 (1)	1 (0)	NA	0	NA
6	(rs6939576=G) or ((rs2859365=G) or (rs6930033=A))	rs2859365	28391465	intergenic	*ZSCAN12*(dist=23921), *ZSCAN23*(dist=8967)	0 (0)	4 (2)	0 (0)	NA	3	Whole Blood (*TRIM27, ZNF187, ZNF193*); Lymphoblastoid (*ZSCAN23*)
6	(rs6939576=G) or ((rs2859365=G) or (rs6930033=A))	rs6930033	29323905	exonic	*OR5V1*	0 (0)	4 (0)	1 (0)	NA	6	Whole Blood (*TRIM27*)
6	(rs7773193=C) or ((rs17280818=T) and (rs2394100=T))	rs7773193	28611334	intergenic	*ZBED9*(dist=56222), *LINC01623*(dist=216068)	31 (4)	6 (0)	24 (0)	A549 (USF1); GM12878 (TBP); H1-hESC (TBP); HEK293(b) (KAP1); HeLa-S3 (AP2GAMMA, BRCA1, CEBPB, RFX5, RPC155, STAT1, TBP, TFIIIC110); HepG2 (CEBPB, HSF1, TBP); K562 (RPC155, TBP, TFIIIC110)	2	NA
6	(rs7773193=C) or ((rs17280818=T) and (rs2394100=T))	rs17280818	28697751	intergenic	*ZBED9*(dist=142639), *LINC01623*(dist=129651)	7 (1)	65 (19)	100 (21)	NA	2	Whole Blood (*ZFP57*)
6	(rs7773193=C) or ((rs17280818=T) and (rs2394100=T))	rs2394100	28422906	intergenic	*ZSCAN23*(dist=11627), *GPX6*(dist=48167)	0 (0)	2 (0)	3 (0)	NA	0	NA
7	((rs12671658=T) or (rs12702656=A)) and (rs11768586=G)	rs12671658	7842281	intronic	*UMAD1*	1 (0)	6 (0)	2 (0)	NA	7	NA

Chr	3-SNP Haplotype	SNP	Chr position (hg19)	Ontology	Mapped Gene	DHS overlap[a], # EIDs (PBC EIDs)	H3K4me1 overlap[a], # EIDs (PBC EIDs)	H3K4me3 overlap[a], # EIDs (PBC EIDs)	Bound protein[b]: Cell line (protein)	# Altered motifs[c]	Significant eQTLs[d]: Tissue (gene)
7	((rs12671658=T) or (rs12702656=A)) and (rs11768586=G)	rs12702656	7851742	intronic	*UMAD1*	0 (0)	9 (3)	0 (0)	NA	4	NA
7	((rs12671658=T) or (rs12702656=A)) and (rs11768586=G)	rs11768586	7849806	intronic	*UMAD1*	1 (0)	6 (2)	1 (1)	NA	0	NA
9	(rs4979484=C) or ((rs13300483=T) and (rs7028891=G))	rs4979484	117751450	intergenic	*TNFSF8*(dist=58575), *TNC*(dist=30404)	22 (6)	33 (20)	11 (7)	GM12878 (BATF, NFKB); GM12891 (NFKB); GM15510 (NFKB); GM18951 (NFKB); HeLa-S3 (AP2GAMMA, BAF155, CEBPB, CJUN, GTF2F1, JUND, P300, RAD21, RFX5, STAT3)	3	NA
9	(rs4979484=C) or ((rs13300483=T) and (rs7028891=G))	rs13300483	117643362	intergenic	*TNFSF15*(dist=74954), *TNFSF8*(dist=12261)	0 (0)	11 (6)	2 (0)	NA	2	Whole Blood (*TNFSF8*)
9	(rs4979484=C) or ((rs13300483=T) and (rs7028891=G))	rs7028891	117645015	intergenic	*TNFSF15*(dist=76607), *TNFSF8*(dist=10608)	0 (0)	4 (3)	1 (1)	NA	3	Whole Blood (*TNFSF8*)
9	(rs7028891=G) and ((rs4979462=T) or (rs10739402=T))	rs7028891	117645015	intergenic	*TNFSF15*(dist=76607), *TNFSF8*(dist=10608)	0 (0)	4 (3)	1 (1)	NA	3	Whole Blood (*TNFSF8*)
9	(rs7028891=G) and ((rs4979462=T) or (rs10739402=T))	rs4979462	117567013	intronic	*TNFSF15*	36 (3)	79 (8)	31 (2)	ECC-1 (ERALPHA_A); HUVEC (CFOS, GATA2); HeLa-S3 (ELK4); HepG2 (FOXA1); T-47D (ERALPHA_A, FOXA1, GATA3)	3	NA
9	(rs7028891=G) and ((rs4979462=T) or (rs10739402=T))	rs10739402	116873231	intergenic	*KIF12*(dist=11894), *COL27A1*(dist=44594)	1 (1)	22 (1)	1 (0)	NA	3	NA
9	(rs7028891=A) and ((rs2418376=A) or (rs10759773=G))	rs7028891	117645015	intergenic	*TNFSF15*(dist=76607), *TNFSF8*(dist=10608)	0 (0)	4 (3)	1 (1)	NA	3	Whole Blood (*TNFSF8*)
9	(rs7028891=A) and ((rs2418376=A) or (rs10759773=G))	rs2418376	118338852	intergenic	*DEC1*(dist=173929), *LOC101928775*(dist=163097)	0 (0)	0 (0)	0 (0)	NA	1	NA
9	(rs7028891=A) and ((rs2418376=A) or (rs10759773=G))	rs10759773	118136224	intronic	*DEC1*	8 (0)	34 (1)	9 (0)	NA	7	NA
9	(rs10817678=G) or ((rs1407306=T) and (rs7048742=A))	rs10817678	117579457	intergenic	*TNFSF15*(dist=11049), *TNFSF8*(dist=76166)	0 (0)	4 (0)	2 (0)	NA	11	Whole Blood (*TNFSF15, TNFSF8*)
9	(rs10817678=G) or ((rs1407306=T) and (rs7048742=A))	rs1407306	117586409	intergenic	*TNFSF15*(dist=18001), *TNFSF8*(dist=69214)	1 (0)	23 (2)	3 (0)	NA	9	Whole Blood (*TNFSF8*)
9	(rs10817678=G) or ((rs1407306=T) and (rs7048742=A))	rs7048742	117441568	intronic	*LOC100505478*	0 (0)	33 (2)	10 (1)	NA	0	NA

Chr	3-SNP Haplotype	SNP	Chr position (hg19)	Ontology	Mapped Gene	DHS overlap[a], # EIDs (PBC EIDs)	H3K4me1 overlap[a], # EIDs (PBC EIDs)	H3K4me3 overlap[a], # EIDs (PBC EIDs)	Bound protein[b]: Cell line (protein)	# Altered motifs[c]	Significant eQTLs[d]: Tissue (gene)
9	(rs4979462=T) or ((rs10739402=T) and (rs10817564=C))	rs4979462	117567013	intronic	*TNFSF15*	36 (3)	79 (8)	31 (2)	ECC-1 (ERALPHA_A); HUVEC (CFOS, GATA2); HeLa-S3 (ELK4); HepG2 (FOXA1); T-47D (ERALPHA_A, FOXA1, GATA3)	3	NA
9	(rs4979462=T) or ((rs10739402=T) and (rs10817564=C))	rs10739402	116873231	intergenic	*KIF12*(dist=11894), *COL27A1*(dist=44594)	1 (1)	22 (1)	1 (0)	NA	3	NA
9	(rs4979462=T) or ((rs10739402=T) and (rs10817564=C))	rs10817564	116827079	intronic	*AMBP*	0 (0)	2 (2)	2 (0)	NA	2	NA

[a] Counts of the number of consolidated cell types (EIDs) for which the SNP of interest overlaps the queried epigenomic assay peak (Roadmap Epigenomics Mapping Consortium processed data, Kundaje et al.[21]). "PBC EID": Separately considers peak overlap counts among the 29 blood/liver cell types available in Roadmap Epigenomics.
[b] Bound protein: Regulatory protein-binding ChIP-seq peak overlaps for specified proteins are provided for blood- or liver-related cell lines only (HaploReg v4, Ward and Kellis[37]).
[c] Altered motifs: The number of regulatory motifs predicted to be affected by the SNP based on position weight matrices (PWM) score changes (HaploReg v4, Ward and Kellis[37]).
[d] eQTLs: Reported significant eQTLs for whole blood, lymphoblastoid, and liver cell types only (GTEx Consortium[20]; HaploReg v4, Ward and Kellis[37]).
Abbreviations: EID, epigenome identifier; dist, distance; #, number; DHS, DNase I hypersensitivity site; eQTL, expression quantitative trait loci.
